# Modelling of acid brown 14 and acid yellow 36 dyes adsorption from water by self-nitrogen-doped activated carbon

**DOI:** 10.1038/s41598-025-14124-7

**Published:** 2025-08-18

**Authors:** Mohamed A. El-Nemr, Mohamed A. Hassaan, Murat Yılmaz, Ahmed El Nemr

**Affiliations:** 1 Department of Chemical Engineering, Faculty of Engineering, Minia University, Minia, 61519 Egypt; 2 The Higher Canal Institute of Engineering and Technology, Al Salam 1 - Abu Bakr Al Siddiq Street, Suez, Egypt; 3 Environment Division, National Institute of Oceanography and Fisheries (NIOF), Kayet Bey, Elanfoushy, Alexandria Egypt; 4 Department of Chemistry and Chemical Processing Technologies, Bahçe Vocational School, Osmaniye Korkut Ata University, 80000 Osmaniye, Turkey

**Keywords:** Self-nitrogen-doped activated carbon, Azo dye adsorption, Fish waste-derived adsorbent, Langmuir isotherm model, Response-surface methodology (RSM), Environmental water treatment, Environmental chemistry, Chemical engineering

## Abstract

Acid Brown 14 (AB14) and Acid Yellow 36 (AY36) are synthetic azo dyes extensively utilized in numerous industries, resulting in detrimental environmental consequences. This study aims to manufacture self-nitrogen-doped porous activated carbon (AC7-800) and investigate its effectiveness in removing the AB14 and AY36 dyes from water solutions. The AC7-800 was created by combining fish waste (with a protein composition of 60% as a nitrogen source), which served as a self-nitrogen dopant. An equal mass ratio (1:1:1) of sawdust, fish waste, and zinc chloride underwent a hydrothermal treatment at 180 °C for 5 h. Subsequently, the material underwent pyrolysis for 1 h in a continuous flow of nitrogen gas at 800 °C to produce AC7-800. The AC7-800 adsorbent was successfully tested and approved to eliminate colours from water in batch trials. The AC7-800 samples were analyzed using BET, SEM, EDX, XRD, FTIR, TGA, and DTA techniques. The results demonstrated the practical synthesis of AC7-800 with a nitrogen mass percentage concentration of 13.73%. The specific surface area, mean pore diameter and monolayer volume were measured to be 437.51 m^2^ g^− 1^, 2.01 nm, and 100.52 cm^3^ g^− 1^, respectively. The objective is to examine the elimination of AB14 and AY36 dyes from a water-based solution using various factors such as initial dye concentration, solution pH, AC7-800 dosage, and contact time. The efficacy of AC7-800 in removing AB14 and AY36 dyes was found to be dependent on the pH level. The highest elimination efficiency of 63.29% and 85.86% was achieved at pH 1.5 for AB14 and AY36 dyes, respectively. Additionally, the maximum adsorption capacity (*Q*_*m*_) for AB14 and AY36 dyes was determined to be 107.5 and 263.2 mg g^− 1^, respectively. The equilibrium data demonstrated a good association with the Langmuir model (LIM) for both dyes, although the best-fit kinetic model was the pseudo-second-order model (PSOM). Electrostatic interactions between the dye molecules and the charged spots on the AC7-800 surface cause both dyes to adsorb. The prepared AC7-800 can be considered a highly effective, accessible, and environmentally acceptable adsorbent for the adsorption of AB14 and AY36 dyes from simulated water. AB14 and AY36 dyes adsorption to AC7-800 was predicted by the response-surface methodology (RSM) and artificial neural networks (ANN) models. The ANN model was more effective in predicting AB14 and AY36 dyes adsorption than the D-optimal RSM, and it was highly applicable in the sorption process.

**Supplementary Information:**

The online version contains supplementary material available at 10.1038/s41598-025-14124-7.

## Introduction

Water systems now include a variety of complex molecules, hazardous chemicals, synthetic compounds, and organic contaminants due to rapid industrialization, population development, and global warming. This is now a significant issue for humanity. Therefore, by practicing water conservation, employing it judiciously, and purifying it, significant positive impacts can be achieved across all domains. Wastewater from industries is particularly harmful because it contaminates environmental waterways, animal and human drinking water, and utility supplies. Making these liquids reusable and purified is one method to stop this from happening^1,2^.

Industries such as chemicals, metal coating, refineries, food processing, fertilizers, pesticides, plastics, textiles, paper, batteries, cosmetics, and pharmaceuticals frequently employ hazardous dyes. These dyes harm aquatic life, human health, and ecological equilibrium^3–7^. Due to their solubility in water and ability to obstruct light in aquatic environments, textile dyes can pose significant risks to human health, including cancer development. In textile dyes and other sectors, azo dyes are often used^8,9^. Azo dyes contain molecular structures containing (-N = N-) linkages^10^.

Acid Brown 14 (AB14) and Acid Yellow 36 (AY36) dyes are examples of azo dyes^11,12^. AB14 dye in wastewater is derived from several sources, particularly in the textile, leather, and printing processing sectors. Because of their harmfulness, these dye mixtures negatively affect the environment and living things and are almost non-biodegradable^13^. As a result, it is determined that these effluents include an essential amount of toxic aromatic dyes, especially azo dyes. As previously mentioned, one of the most prominent causes of aesthetic contamination and disturbance to the aquatic ecosystem is the colored wastewater released into the environment during the production methods of the textile, leather, and printing processing industries^14^. Acid Yellow 36, another azo dye, is also present in various industrial wastewaters from tanning, paper, soap, textiles, cosmetics, polish, candles, and other industries. This dye is mono-azo. It is carcinogenic and poisonous and has been shown to acutely poison Heteropneustes fossils. Apart from its fatal effects, Acid Yellow 36 dye causes decreased body weight, colour changes, restlessness, jerky movements, and erratic movements in test fish^15,16^.

It is crucial to remove and/or recover dyestuffs using the proper techniques since they are hazardous, carcinogenic, and can pose a significant threat to human and environmental health. The methods used for dye elimination for years can be listed as adsorption^17,18^, coagulation/flocculation^19,20^, membrane filtration^21,22^, oxidation procedures (photocatalysis)^23,24^, ion exchange^25,26^, reverse-osmosis^27,28^ biological treatment and biological processes^29,30^, chemical precipitation^31^, and advanced oxidations^32,33^, etc.

These methods have several drawbacks, including high costs, low efficiency, additional waste products, etc. Conversely, adsorption has several benefits as a substitute technique, including low cost, low energy consumption, straightforward design, user-friendliness, high efficiency, and adsorbent reuse^34,35^.

It is considered a wise decision, particularly when employing inexpensive, safe, and ecologically friendly adsorbents that can lessen dyestuffs. Nonetheless, commercially activated carbons are regarded as costly due to their relatively expensive and nonrenewable raw ingredients, which are unsuitable for pollution prevention applications^36^. Substitute low-cost materials that do not require additional expensive treatment to make the adsorption process practical and affordable. Adsorbents must be able to adsorb substances, but a steady supply of the precursor adsorbent is also necessary for an effective adsorption process. As a result, using inexpensive precursors (industrial, animal, mineral, or agricultural waste) is advised. Among these precursors, different natural materials and agricultural biomasses have been utilized for manufacturing activated carbon, such as fly ash^37^, coconut shells^38^, mandarin peels^39^, nutshell^40^, date palm petiole^41^, cocoa pods^42^, maize tassel^43^, coffee waste^44^, sawdust^45^, and algae^46^. This investigation was spurred by the hunt for a novel precursor and examined a combination of sawdust and fish waste.

Sawdust, a byproduct of wood processing, is utilized in manufacturing furniture and construction applications. When placed around plants and shrubs, sawdust preserves moisture, inhibits the growth of weeds and grass, and cools the roots of the plants. According to Park et al.^47^, sawdust can be used to grow cucumber and tomato plants. Sawdust was also demonstrated to be used as an additive in manufacturing hollow concrete by Adebakin and Adeyemi^48^. Shukla et al.^49^ provided evidence of sawdust’s function in filtering out undesirable substances from water. Sawdust has benefits, but disposing of it could have adverse environmental effects. Sawdust has low ash but high carbon content (50% w/w)^50^. Angela et al.^51^ reported that sawdust has an ash content as low as 0.08% and a carbon content ranging from 77.51 to 93.59%.

Fish industry biowaste products have garnered much interest as unique, unprocessed substances that can be utilized for various purposes. During fish processing, 50 to 75% of fish and seafood by-products, such as fins, viscera, scales, bones, shells, skin, and flesh, are discarded as waste^52,53^. Given that the predicted global fish production in 2019 was 177.8 million metric tons (a figure expected to rise steadily), this waste occurs in enormous amounts^54^. Approximately 7.2–12 million tons of this total are squandered yearly^55^. These waste materials are dumped into the environment, landfills, or the ocean, causing significant financial loss and harm to aquatic ecosystems, odour, and greenhouse gas emissions^56–58^. Organic matter is increased in the ocean via the disposal of fish feces.

Nitrogen doping is a highly successful method to increase the adsorption efficacy of activated carbon. It increases the electrical conductivity, increases the number of ion-storage sites, and decreases the amplitude due to the presence of nitrogen atoms, which have an extra electron compared to carbon atoms^59–61^. When carbon material is exposed to doped nitrogen atoms, the nitrogen atoms within the hexagonal carbon ring induce localized strain, leading to the distortion of the original carbon structure^62^. According to recent research, doping-activated carbons with a specific concentration of phosphorus, sulphur, or nitrogen increases part of their pseudo capacitance and improves surface stability, increasing the particular capacity of the carbons overall^63–65^. Additionally, an N-doped carbon nanosphere containing metal was employed as a photocatalyst, enabling novel approaches to environmental remediation. One practical way to combat pollution and energy scarcity is by photocatalysis using solar energy^66^.

The RSM, which relies heavily on statistical principles and methods, comprises four main stages: (i) designing experiments, (ii) developing response surface models via regression, (iii) optimizing process parameters, and (iv) validation^67–69^. Conversely, the ANN is a class of machine learning algorithms inspired by the structure and function of the human brain’s biological neurons. It recognizes patterns, interprets data, and develops interconnected computer models within hidden layers to determine complex nonlinear relationships between input and output variables^70^. Using RSM with ANN can be a powerful tool for optimizing AB14 and AY36 dye adsorption using AC7-800.

Although there have been several reports on the integration and comparison of RSM and ANN methodologies in predicting and optimizing process variables, for instance, in optimizing the preparation of magnetic activated carbon for the adsorption of crystal violet^71^, in comparing the performance of KOH and NaOH catalysts for biodiesel production^72^, and in predicting and optimizing salicylic acid removal by activated carbon^73^, no investigation has so far explored D-optimal designs and ANN model in predicting AB14 and AY36 dyes adsorption using AC7-800 during an in-depth literature search.

This work aims to evaluate the consequences of removing AY36 and AB14 dyes from water by specifically examining and detailing a distinctive process for producing activated carbon with high nitrogen content through self-doping with nitrogen, utilizing ZnCl_2_ as the activating agent. In this study, activated carbons have been made by thermal treatment of sawdust, fish waste, and ZnCl_2_ to remove AB14 and AY36 dyes from the aqueous solution. Kinetic, isotherm, RSM and ANN modelling have been studied for the adsorption of AB14 and AY36 dyes using prepared self-doping with nitrogen AC7-800.

## Materials and methods

### Chemicals and equipment

**Fig. 1:**
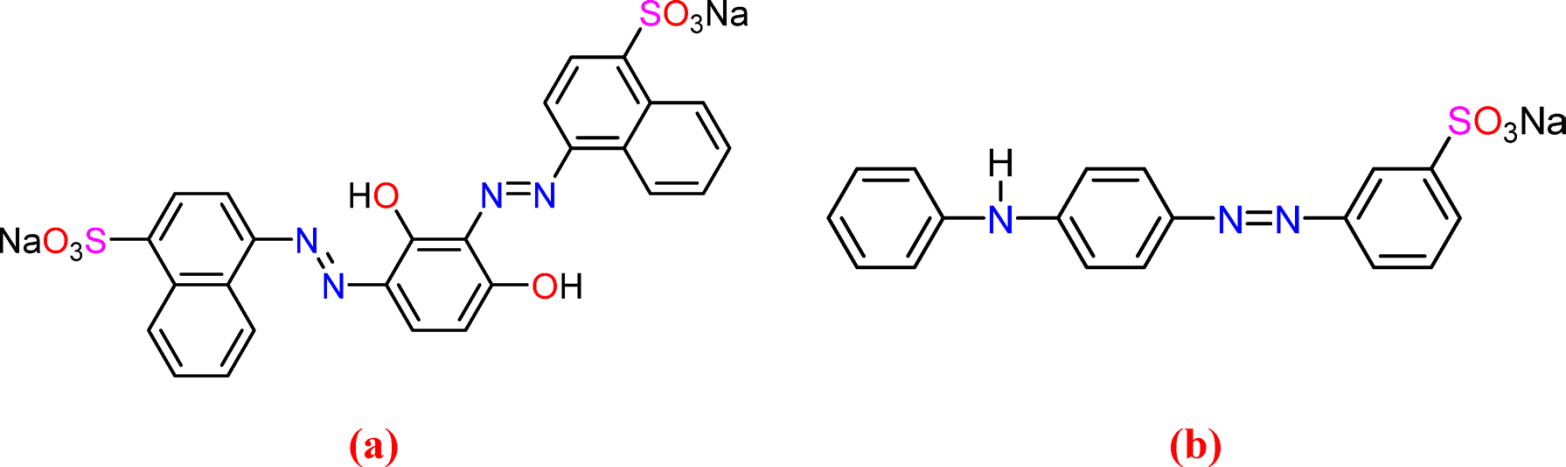
Chemical and molecular structures of (**a**) AB14 dye and (**b**) AY36 dye.

Sawdust was supplied by a carpenter in Alexandria, Egypt, and dried ground fish waste (60% protein) was provided by fishermen in Alexandria, Egypt. The molecular weight of hydrochloric acid (HCl) is 36.46, and it was acquired by SD Fine-Chem Limited (SD FCL), located in Mumbai, India. Based on acid-metric analysis, the acid has an assay range of 30–34%. Ethanol (C_2_H_5_OH) and Zinc chloride (ZnCl_2_) were purchased from Sigma Aldrich Company. Nitrogen (N_2_) gas was purchased from a local supplier in Alexandria, Egypt. Acid Brown 14 (AB14) dye (C.I. 22110) (C_26_H_16_N_4_Na_2_O_8_S_2_) (Mwt = 622.547 g/mol) and Acid Yellow 36 (AY36) dye (Orange II) (C.I. 13065) (C_18_H_16_N_3_NaO_3_S) (Mwt = 375.38 g/mol) were obtained from ISMA Dye company, Egypt. Figure 1 shows the chemical and molecular structure diagrams of AB14 and AY36 dyes. All experimental studies utilized double-distilled water.

The dye concentration was measured using a UV-visible spectrophotometer (Analytic Jena, model SPEKOL1300) with glass cells with an optical path length of 10 mm. The experiment was carried out with a Nabertherm B180 Tubular Furnace (RT 50/250/13) from Germany, a JENCO pH meter (model 6173), and a JS shaker (model JSOS-500). The adsorbent surface was analyzed using Fourier Transform Infrared (FTIR) spectroscopy, specifically the platinum attenuated total reflection (ATR) model V-100 connected to Bruker VERTEX70 equipment, Germany. This method facilitated identifying surface functional groups across a wavenumber range of 400 to 4000 cm^− 1^. The elemental composition and surface morphology of biochar samples were examined using a Scanning Electron Microscope (SEM) (QUANTA 250) that was equipped with an Energy Dispersive X-ray Spectrometer (EDX). The SDT650-Simultaneous Thermal Analyzer instrument was utilized to perform thermal assessments. The device has a temperature range from ambient to 1000 °C, with a heating rate of 5 °C per minute. The X-ray Photoelectron Spectroscopy (XPS) analysis was conducted utilizing a Thermo Fisher Scientific K-Alpha XPS instrument. The pass energy used was 50 electron volts (eV), and the study was completed at a base pressure of around 10^–9^ millibar (mbar).

The boiling point of N_2_ gas was used to quantify the nitrogen gas adsorption-desorption isotherm on activated carbon. The activated carbon’s BET surface area (*S*_*BET*_) was ascertained using N_2_ adsorption measurements at 77 K utilizing the BELSORP – Mini II analyzer produced by BEL Japan, Inc. A BET isotherm study was performed to determine the surface area (*S*_*BET*_) in m^2^ g^–1^, monolayer volume (*V*_*m*_) in cm^3^ (STP) g^–1^, energy constant (*C*), total pore volume (*V*_*T*_) in *p*/*p*_0_ cm^3^ g^–1^, and mean pore diameter in nm. Equation (1) was used to get the average pore radius.

1 $$r\left( {nm} \right)=\frac{{2{V_T}\left( {{\text{mL}}\;{{\text{g}}^{ - 1}}} \right)}}{{{a_{s,BET}}\left( {{{\text{m}}^2}\;{{\text{g}}^{ - 1}}} \right)}} \times 1000$$

The Barrett–Joyner–Halenda (BJH) methodology was utilized to calculate the micropore volume (*V*_*mi*_) and micropore surface area (*S*_*mi*_) of activated carbon, as well as the mesopore surface area (*S*_*mes*_) and mesopore volume (*V*_*mes*_). These measurements were obtained using the BELSORP analysis software. The pore size distribution is ascertained using the BJH method applied to the desorption isotherm.

### Preparation of N-doped activated carbon (AC7-800)

N-doped activated carbon was synthesized using sawdust as a starting material, and fish waste (60% protein) was ground hydrothermally treated at 180 °C, followed by pyrolysis under nitrogen at 800 °C. Mix 50 g sawdust with 50 g ground Fish waste (60% protein), 50 g ZnCl_2_, and 300 mL distilled H_2_O. The combination underwent hydrothermal treatment at 180 °C for 5 h in a 500 mL Teflon cup within a stainless steel autoclave. The hydrothermal treatment product was thoroughly mixed with 50 g of sawdust in a mortar. The mixture was then dried at 125 °C in an oven for one night. Then, the dried product was burned at carbonization temperatures of 800 °C (following our previous carbonization published work^34,74]– [75^ under N_2_ for 1 h to give the primary activated carbon. The primary activated carbon was filtered and refluxed for 2 h in 2 N HCl. The activated carbon that had undergone reflux was subjected to filtration, followed by washing with water and ethanol. Afterward, it was dehydrated overnight at 125 °C. Sonication was performed for refluxed activated carbon under 100 mL H_2_O for 30 min, decanted, washed with 100 mL of ethanol, and then subjected to drying at a temperature of 125 °C in a furnace for one night. N-doped activated carbon was obtained and labelled as AC7-800.

### Determination of zero-point charge (pH_PZC_)

The pH_PZC_ was obtained using the technique described in the literature^76,77^. Briefly, AC7-800 (50 mg) was suspended in 0.1 M NaNO_3_ solution (50 mL) in a 100 mL flask. After adjusting the original pH solution (pH_i_) with 0.1 M HCl or NaOH to a value between 2 and 12, it was shaken for 24 h. Next, the supernatant liquid’s final pH (pH_f_) was determined. A variance plot against pHi was also made for the initial and final pHs (ΔpH = pH_i_ − pH_f_). The adsorbent’s pH_PZC_ was determined by measuring the pH value at which ΔpH equalled zero. According to reports, the pH_PZC_ value of AC7-800 was 9.60. The result suggests that the nitrogen atoms in AC7-800-H^+^ undergo protonation and have left the surface of the AC7-800 positively charged below this pH threshold (9.60).

### Adsorption experiments (adsorption of AB14 and AY36 Azo dyes)

The adsorption experiments were conducted separately for each dye, where a concentration of 1000 mg L^–1^ was achieved by dissolving 1.0 g of each dye in 1000 mL of double-distilled water to create a stock solution. The batch equilibrium approach was employed to investigate AB14 or AY36 dye adsorption. A volume of 100 mL of the solution containing the substance to be adsorbed with different initial dye concentrations was mixed with varying amounts of the AC7-800 used for adsorption in a shaker (JSOS-500). The remaining dye concentration was investigated utilizing a visible-UV spectrophotometer at an adsorption wavelength of *λ*_max_ = 594 nm for AY36 dye or *λ*_max_ = 461 nm for AB14 dye. Equation (2) can be used to quantify the adsorption capabilities of AC7-800.

2 $${q_t}=\frac{{\left( {{C_0} - {C_t}} \right)}}{w} \times V$$

where the variable *C*_*0*_ represents the initial concentration of the contaminant (mg L^–1^), *q*_*t*_ denotes the adsorbent’s adsorption capacity (mg g^–1^) at a specific time *t*. *C*_*t*_ (mg L^–1^) denotes the remaining concentration of the pollutant after it has been adsorbed for a particular period *t*. *V* (L) refers to the volume of the dye solution in liters, and *W* (g) represents the mass of the adsorbent AC7-800 in grams. Equation (3) determines the proportion of dye removed from an aqueous solution.

3 $$Removal\;~\left( \% \right)=~\frac{{{C_o} - {C_t}}}{{{C_o}}} \times 100$$

### Studies on parameters affecting adsorption

An investigation was conducted to investigate the pH impact of eliminating dyes. Fifty milligrams of the adsorbent were introduced into 100 mL of AB14 and AY36 dyes, with initial pH values adjusted across a range of 1.5 to 11 for both dyes. The concentration of the dye solutions was 100 mg L^–1^. The pH was modified using a 0.1 M NaOH or a 0.1 M HCl solution. After two hours of mixing at 200 rpm at room temperature, the suspensions were collected to determine the remaining dye.

The isotherm analysis employed various amounts of dye solution of starting concentrations (100–400 mg L^–1^) for AC7-800. Multiple concentrations of AC7-800 (50–250 mg) were mixed with 100 mL solutions containing various starting dye concentrations (100–400 mg L^–1^). The mixtures were agitated at a velocity of 200 rpm for 120 min at 25 °C.

The impact of the amount of adsorbent used on the elimination of dye was investigated by agitating 100 mL of dye solutions with concentrations of 100–400 mg L^–1^, using dosages of AC7-800 at 0.5–2.5 g L^–1^. The agitation was performed for 10, 15, 30, 45, 60, 90, and 120 min at 25 °C.

In the kinetics investigation, Erlenmeyer flasks holding 100 mL of dye starting concentration (100–400 mg L^–1^) were each supplemented with 0.50, 1.00, 1.50, 2.00, and 2.50 g L^–1^ of AC7-800. The suspensions were stirred at a speed of 200 rpm at 25 °C. At regular solution intervals (10, 15, 30, 45, 60, 90, and 120 min), samples were collected to analyze the remaining color residue. All experimental work was performed in triplicate, and the standard error of these three replicates was ≤2.1, so we used the average values ​​across all studies throughout the manuscript. All experiments were performed three times and only the mean values were used in figures and modelling where the standard deviation was ≤2.14.

### Optimization study response surface methodology

The traditional design of experiment techniques (Box-Behnken, Factorial Fractional, Full Factorial Designs, and RSM Designs) helped standardize standardizing the linear model in experimental contexts where parameters are largely unrestricted in the interest domain. Nonlinear models, on the other hand, are inescapable in some situations. Some solutions (factor level combinations) can be excessively complex or difficult to estimate in some circumstances. Model-specific designs that solve standard design constraints are D-optimal designs^78,79^. An iterative search procedure yields a D-optimal design to reduce the parametrical covariance calculation for a specific design. This is similar to the maximization of the determining factor D = [XTX], while X is the design matrix of model terms assessed at different handlings in the design space. Unlike conventional designs, D-optimal designs do not need the orthogonal design^80^.

The D-optimal design to study the adsorption of AB14 and AY36 dyes from water was analyzed using the State-Ease Design Expert v 13.0.5.0 software^81^. The optimization of effective parameters in the adsorption process, specifically the influence of three independent variables (A: AC7-800 dosage, B: starting concentration of dye, and C: time of contact) on the response variable (R: dye removal %), was investigated utilizing RSM.

A statistical method called RSM uses quantitative data from pertinent experiments to determine the operational parameters and regression model equations. Three steps comprise the optimization process: carrying out a statistically planned experiment, figuring out the mathematical model’s coefficients, forecasting the response variable, and assessing how well the resulting model works^82^. The experiment’s range and variables are presented in Table 1.

**Table 1:** Levels and ranges used in the batch desorption investigation.

Factor	A	B	C
Name	AC7-800 dosage	Dye Conc.	Time
Units	mg	mg/L	min
Type	Numeric	Numeric	Numeric
Sub Type	Discrete	Discrete	Discrete
Minimum	50	100	15
Maximum	250	400	120
Coded Low	− 1 ↔ 50.00	− 1 ↔ 100.00	− 1 ↔ 15.00
Coded High	+ 1 ↔ 250.00	+ 1 ↔ 400.00	+ 1 ↔ 120.00
Mean	132.50	262.50	66.75
Std. Dev.	81.56	128.63	44.99

Six axial points, eight factorial points, and six repeats at the central point were used to determine the optimal custom design for the three independent variables. Five levels (–α, − 1, 0, 1, +α) of manipulation were applied to the selected variables. The number of experimental trials was determined using Eq. (4):

4 $$N\,=\,{{\text{2}}^{\text{k}}}\,+\,{\text{2}}.{\text{k}}\,+\,{\text{C}}\,=\,{{\text{2}}^{\text{3}}}+{\text{ 2}}.{\text{3}}\,+\,{\text{6}}\,=\,{\text{2}}0$$

where *C* is the number of experiments conducted at the centre, *N* is the number of runs, and k is the number of parameters to be assessed. The lowest and highest values for each component are shown in Table 1. State-Ease Design Expert 13.0.5.0 was used to create the experimental data matrix. ANOVA was used to statistically analyze the obtained model. The relationships among variables were analyzed by surface contour plots.

### Artificial neural networks (ANN) modelling

**Fig. 2:**
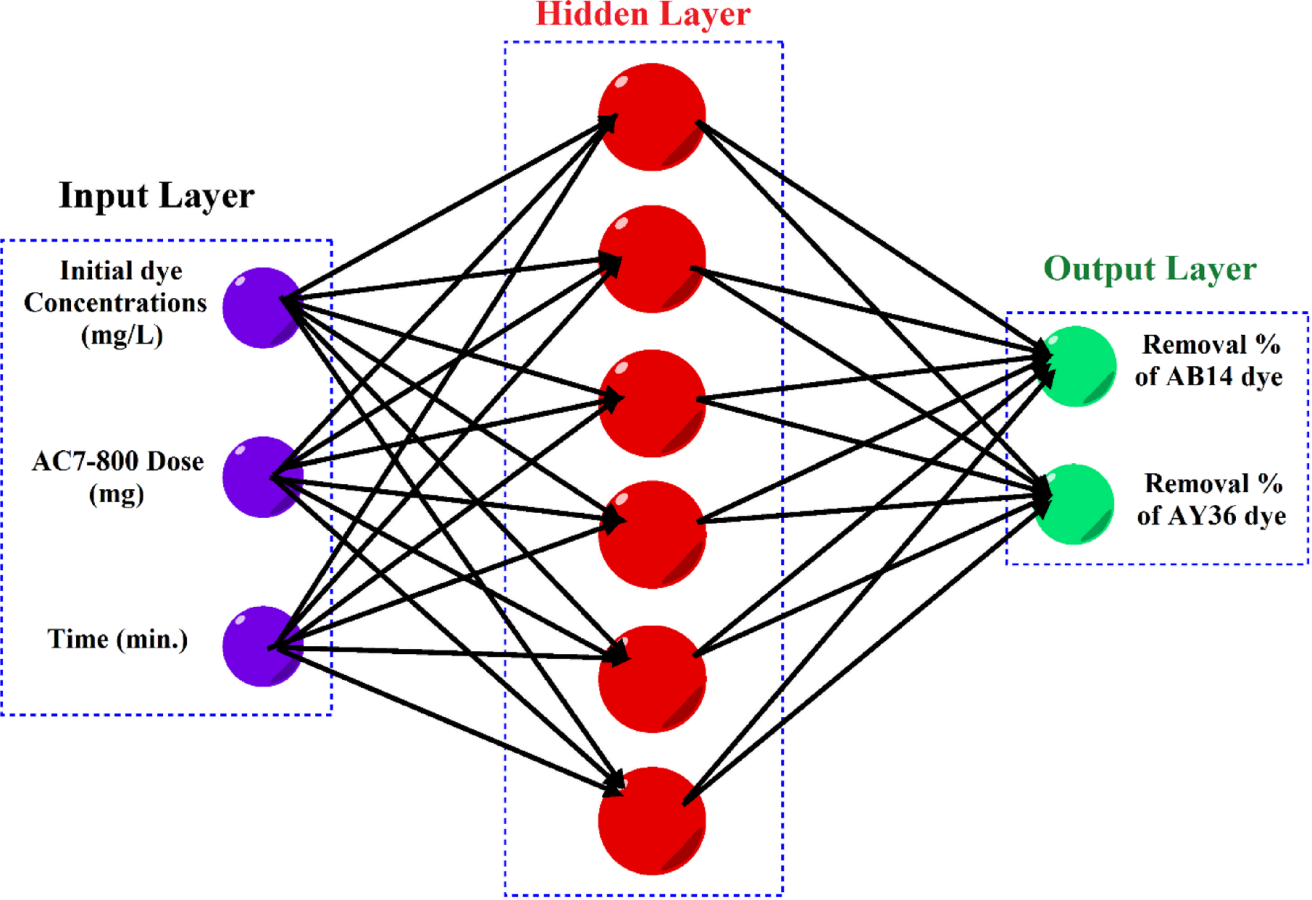
ANN net (input, hidden, and output layers) was utilized in this study.

ANN modelling software simulates the biological human brain networks by predicting complex linear and nonlinear relationships between input and output data sets. The neuron was the main building unit of the ANN. The neurons store and process large amounts of data in the ANN architecture. The most common type of ANN was the feed-forward back-propagation NN (BPNN). The components of the BPNN are an input layer (IL) (independent variable), hidden layers (HNs), and an output layer (OL) (dependent variable). The HN is a vital component of the ANN thanks to its flexibility and adaptability. The ANN approach in this study displayed the removal of Acid Brown 14 (AB14) and Acid Orange 7 (AO7) dyes by AC7-800 using MATLAB R2015b version. The training algorithm of this ANN approach was the Levenberg Marquart (LM) with training (70%), validation (15%), and testing (15%). The efficiency of the ANN model is measured by the correlation coefficient (*R*^2^) and mean square error (MSE). The most efficient network (best-fit ANN model) must show the highest *R*^2^ and the lowest MSE. The best fit ANN model architecture was the BPNN with a hidden layer (HL) of 6 neurons, thanks to its ideal performance. This performance possessed the highest *R*^2^ and the lowest MSE values. The training stage tested 4–10 neurons. Finally, the independent variables were the adsorbent dosage of the AC7-800 (mg), time (min), and initial dye concentration (mg/L). The removals of AY36 and AB14 dyes were the dependent variables^83,84^ (Fig. 2).

## Results and discussion

### Characterization

#### Thermogravimetric analysis

**Fig. 3:**
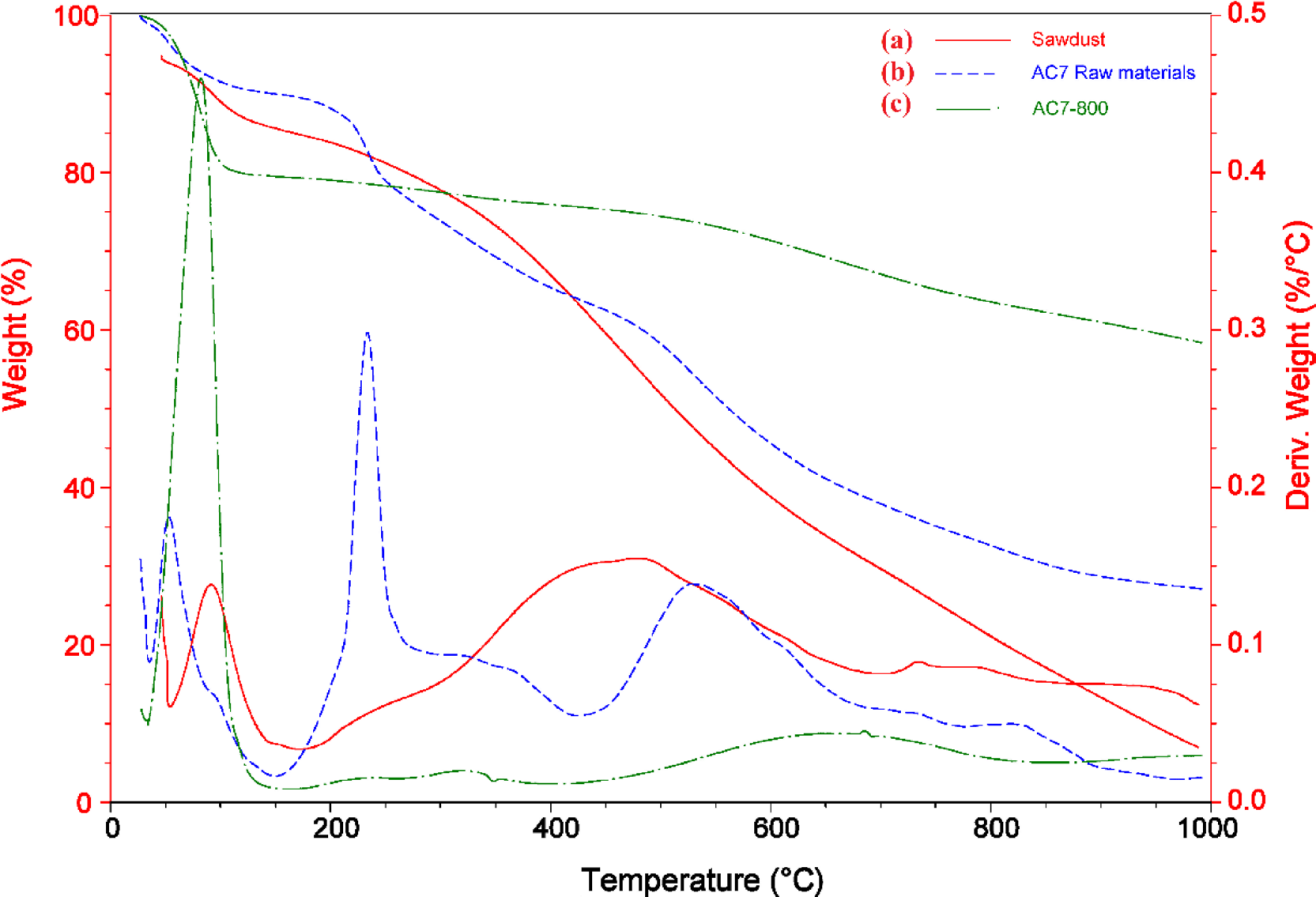
TGA and DTA analyses of (**a**) Sawdust, (**b**) 1:1:1, a mass ratio of sawdust: fish waste: ZnCl_2_, and (**c**) prepared AC7-800.

TGA and DTA analyses were performed on sawdust, a mixture of sawdust, fish waste (with 60% protein content), and ZnCl_2_ with a mass ratio of 1:1:1, and AC7-800 was prepared. The mixture was hydrothermally processed at 180 °C for five hours. Figure 3a illustrates the multistage thermochemical breakdown of sawdust that was found. The initial weight loss event, seen within the 60.39–190 °C range, can be ascribed to removing lignocellulosic material. The temperature between 190 and 714 °C exhibits a secondary mass-loss of 55.15%, with the highest mass-loss occurring at 443.85 °C. The occurrence of volatile cellulosic particles and the degradation of cellulose could account for the observed 22.83% reduction in weight, which took place between temperatures of 714 and 980 °C. The highest magnitude of weight loss was documented at a temperature of 790.25 °C. The standard Fig. 3b displays the exemplary Thermogravimetric Analysis (TGA) and Differential Thermal Analysis (DTA) curves of the blend of substances that underwent hydrothermal processing for five hours at 180 °C. According to the TGA and DTA data, the central thermochemical breakdowns occur at temperatures between 39.08 and 150.0 °C, 150.0 and 455.16 °C, 455.16 and 700 °C, and 700 and 990 °C. These ranges also show weight losses of 9.93, 26.06, 25.57, 5.975, and 11.24%, respectively. In the initial step, a slight elevation in the DTA curve was seen, corresponding to moisture evaporation.

Additionally, there was a slight weight reduction within the 39.08–150 °C temperature range, as indicated in the TGA curve. The temperature dropped significantly from 150 to 455.16 °C in the second step, and the DTA curve displayed a prominent peak. A moderate weight loss occurred during the third stage when the temperature rose from 455.16 to 700 °C. At a temperature range of 700–1000 °C, the last stage displayed a weight loss of 11.24%, which could have been caused by the release of ZnCl_2_ (which has a melting point of about 283 to 290 °C and a boiling point of 732 °C) together with a few volatile compounds. Figure 3c reports the DTA and TGA analyses of the prepared AC7-800. In the initial stage, within the 25–150 °C range, approximately 20.48% weight loss, as indicated on the TGA curve, was observed alongside prominent peaks on the DTA curve, attributed to moisture release. In the subsequent stages, a weight loss of 3.58% occurred between 150 and 400 °C, followed by a further loss of 17.56% between 400 and 1000 °C, with multiple peaks evident on the DTA curve.

#### Textural and morphological properties

**Fig. 4:**
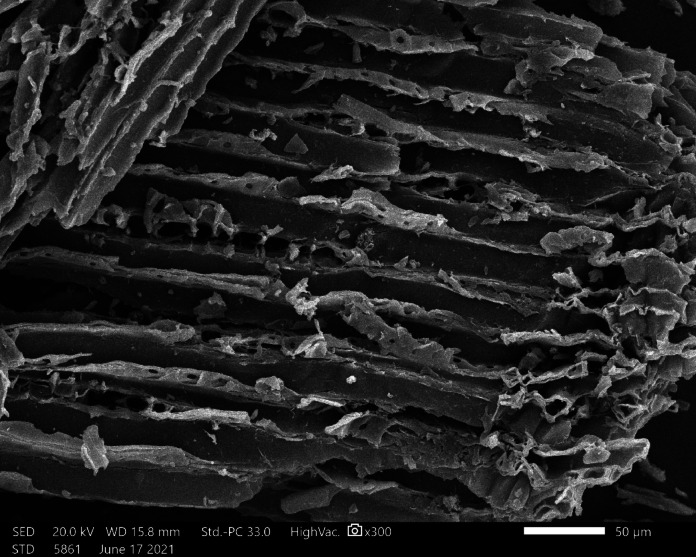
SEM analysis image of AC7-800.

Figure 4 displays the SEM image of AC7-800, revealing a highly porous structure with visible, well-developed channel pores and a uniform pore size distribution. This morphology, formed through high-temperature pyrolysis at 800 °C, facilitates efficient adsorption by providing ample surface area and interconnected pathways for dye molecules to diffuse into the material. The uniform distribution of pores ensures consistent adsorption performance, while nitrogen doping from fish waste introduces active sites that enhance electrostatic interactions with dye molecules. These characteristics collectively contribute to the high adsorption capacity and efficiency of AC7-800 for removing AB14 and AY36 dyes, making it a promising material for wastewater treatment applications.

**Fig. 5:**
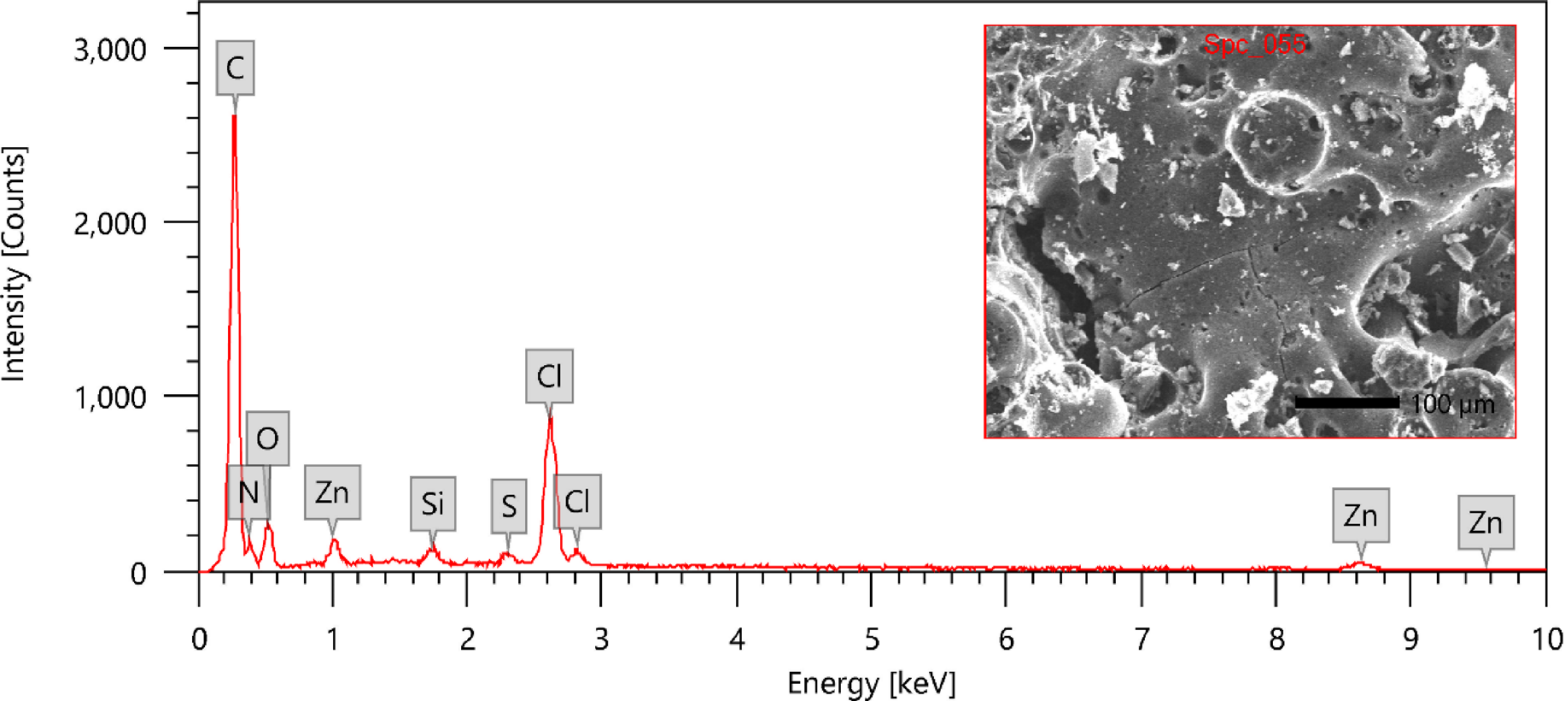
An EDX analysis of prepared AC7-800.

Approximately 60% of fish waste consists of a protein mostly made of C, H, N, and O elements, which can be broken down through hydrolysis at elevated temperatures. Generating AC7-800 comprises eliminating non-carbon components and reorganizing the carbon structure by incorporating nitrogen atoms into the carbon network. This process is achieved through hydrothermal heating at a temperature of 180 °C, followed by pyrolysis in the presence of N_2_ gas flow at 800 °C. As an impregnation reagent, zinc chloride was enhanced the pores created during the pyrolysis procedure^74^. Zinc chloride (ZnCl_2_) undergoes sublimation, transitioning directly from a solid to a gas, and forms pores when exposed to elevated temperatures. The hydroaromatic structure undergoes a process of dehydrogenation and dehydration when ZnCl_2_ reacts with oxygen in the OH functional groups. As a result, Table 2 illustrates that the AC7-800 has larger carbon contents but relatively low oxygen contents. The EDX examination of AC7-800 revealed that its composition was 65.91% carbon, 13.73% nitrogen, and 12.28% oxygen, with negligible quantities of silicon and sulphur (0.33 and 0.34%, respectively), zinc (1.95%), and chlorine (5.45%) (Table 2; Fig. 5).

**Table 2:** Element analysis of prepared AC7-800 using EDX analysis.

Element	Mass %	Atom %
C	65.91 ± 0.27	73.74 ± 0.31
N	13.73 ± 0.49	13.17 ± 0.47
O	12.28 ± 0.30	10.32 ± 0.25
Si	0.33 ± 0.03	0.16 ± 0.01
S	0.34 ± 0.02	0.14 ± 0.01
Cl	5.45 ± 0.08	2.07 ± 0.03
Zn	1.95 ± 0.12	0.40 ± 0.02
Total	100.00	100.00

The BET analysis of the prepared AC7-800 indicates that the average size of all micropores is less than 2 nm (Table 3). These findings suggested that the N-atoms were effectively incorporated into the activated carbons with the most pronounced microporous framework. Figure S1a–f shows the N_2_ adsorption-desorption isotherms of AC7-800. They concluded that the different nitrogen AC7-800s’ adsorption isotherms are microporous, typical Type I, and as a result, there is a strong surface-adsorbate relationship (Fig. S1a). The specific surface area of AC7-800 was 437.51 m² g^–1^, with a monolayer volume of 100.52 cm³ STP g^–1^, a total pore volume of 0.2202 cm³ g^–1^, and a mean pore diameter of 2.0133 nm, as determined by the BET analysis (Table 3, Fig. S1b). The BJH analysis used adsorption and desorption to ascertain the distribution of mesopore pore size. Figure S1c, d depicts the BJH analysis of the desorption–adsorption of AC7-800. The distribution peak of the AC7-800 is located at 3.1 nm, and its mesopore radius ranges from 2.2 to 9.0 nm (Fig. S1c). The mesopore-specific surface area was 27.728 m^2^ g^–1^, and the integrated pore volume (*V*_*p*_) was 0.0465 cm^3^ g^–1^ in the BJH desorption analysis of AC7-800 (Fig. S1d). The mesopore-specific surface area was 44.516 m^2^ g^–1^, and the integrated pore volume (*V*_*p*_) was 0.0589 cm^3^ g^–1^ in the BJH adsorption analysis of AC7-800. The mesopore-specific surface area of nitrogen-doping activated carbons constituted a small portion of the specific surface, based on the BJH desorption and adsorption data. The correlation between the relative pressure and the adsorption layer thickness was shown by the *t*-plot analysis. Figure S1e depicts the AC7-800 *t*-plot curve. The type II micropores in AC7-800 were revealed by *t*-plot analysis, indicating that its micropore sizes were consistent. According to Fig. S1e, AC7-800 has a pore surface area of 540.71 m^2^ g^− 1^. The average pore diameter of AC7-800 is 0.6726 nm, and its pore volume was measured at 0.1749 cm^3^ g^–1^. Due to its evolution from the *t*-plot, the MP-conclusion plot is highly comparable to it as an analysis tool. The MP-plot cannot produce a smooth curve because of the substantial effects of micropore filling and the chemical variations between the sample surface and standard material. On the other hand, the MP-plot can be used to identify the presence of micropores and the size range in which they are found. Figure S1f depicts the MP-plot of AC7-800, demonstrating the micropores with a diameter ranging between 0.4 and 1.1 nm and a maximal distribution peak at 0.7 nm. For AC7-800, the pore volume (*V*_*P*_) was 0.1887 cm^3^ g^–1^, but the pore surface area (*a*_*1*_*–a*_*2*_) was 480.033 m^2^ g^–1^.

**Table 3:** Surface area analysis of the fabrication AC7-800.

Parameters		AC7-800
*a*_*s*_, BET (m^2^ g^− 1^)	*S*_*BET*_ (m^2^ g^− 1^)	437.51
*V*_*m*_ (cm^3^ g^− 1^)	*V*_*m*_ (cm^3^ (STP) g^− 1^)	100.52
Mean pore diameter	*D*_*P*_ (nm)	2.0133
Total pore volume	*V*_*T*_ (cm^3^ g^− 1^)	0.2202

#### Crystallographic structures

**Fig. 6:**
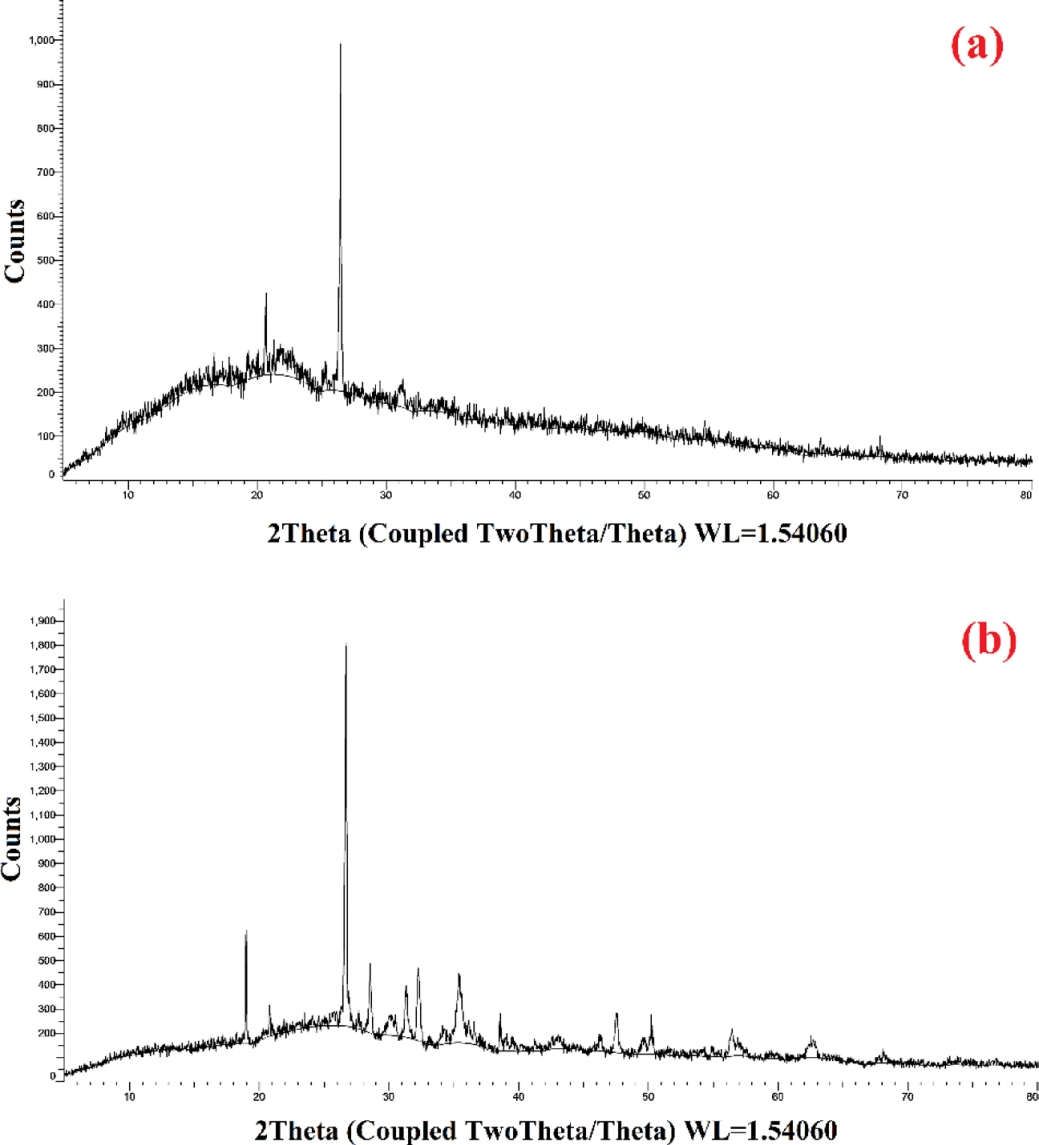
The XRD graph of (**a**) raw material (1:1:1, a mass ratio of sawdust: fish waste: ZnCl_2_), and (**b**) prepared AC7-800.

The fabricated AC7-800’s XRD study was presented in Fig. 6. The X-ray diffraction (XRD) spectra of the raw material and AC7-800 exhibit two peaks at approximately 24° and 44°, corresponding to the (002) and (101) planes of carbon, respectively. Additionally, a peak of lower intensity indicates the presence of smaller crystallites, which is anticipated due to the amorphous nature of the AC7-800.

#### Surface functional groups

**Fig. 7:**
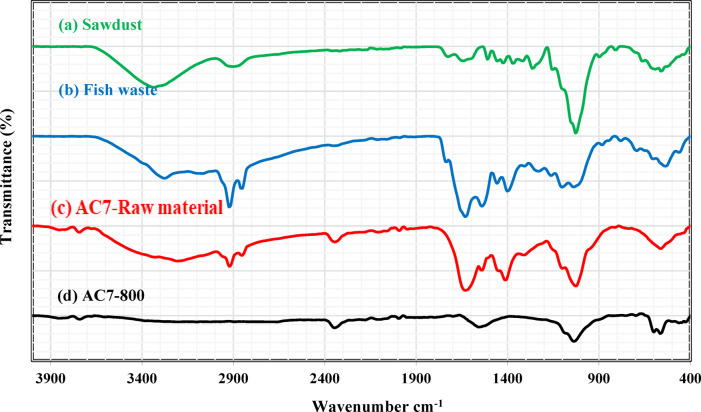
The FTIR figures of (**a**) sawdust, (**b**) Fish waste, (**c**) AC7-Raw material (1:1:1, a mass ratio of sawdust: fish waste: ZnCl_2_), and (**d**) AC7-800.

FTIR analysis was employed to identify the functional groups present in sawdust, fish waste, the combination of sawdust, fish waste, and ZnCl_2_, as well as AC7-800. The findings are displayed in Fig. 7. In the sawdust FTIR spectrum (Fig. 7a), a broad absorption peak is detected at 3324.66 cm^− 1^, which suggests the existence of both unbound and intermolecularly bound hydroxyl (–OH) groups. The peak detected at 1723.59 cm^− 1^ represents the stretching vibrations of –C=O bonds in the aromatic groups of lignin. Conversely, the peak observed at 2899 cm^− 1^ is associated with the stretching vibrations of C–H bonds in the –CH_2_ group. The peak at 1422.97 cm^− 1^ is attributed to the deformation of the –OH group, while the peak at 1634.45 cm^− 1^ may be attributed to the N–H amide group. The C–O stretching of the main alcohol is identified by a distinct and intense peak at 1026.04 cm^− 1^. The peaks at 590.43 and 557.99 cm^− 1^ (Fig. 7a) were identified as the bending vibrational modes of aromatic compounds. Figure 7b displays the FTIR spectrum of the fish waste, which is a combination of Atherina hepseetus and Sardina Pilchardus. For amide A, two broad bands occur at 3279.36 cm^− 1^ and 3066.73 cm^− 1^, respectively. One of these broad bands is attributed to OH, and the other to N–H stretching vibrations. The stretching vibrations of CH_2_ are connected with two distinct amide B bands seen at 2924.18 and 2852.78 cm^− 1^. At 1738.06 cm^− 1^, the ester and lipid groups reach their maxima.

Furthermore, amides I, II, and III have been detected at specific frequencies: 1627.3 cm^− 1^ (corresponding to the stretching vibration of C = O), 1541 cm^− 1^ (associated with the N–H bending and C–N stretching vibrations), and 1308 and 1232.55 cm^− 1^ (related to the N–H bending and C–N stretching vibrations and O = C–N), respectively. The absorption peaks observed at approximately 1104.31, 1040.41, 603.61, and 533.73 cm^− 1^ can be conclusively linked to the stretching of the asymmetric phosphate group (PO_4_^3−^). The FTIR analysis of the mixture of fish waste, raw sawdust, and ZnCl_2_ was illustrated in Fig. 7c. The O-H stretching peak for alcohol and the N-H stretching frequencies were observed at 3337.00 and 3218.61 cm^− 1^, respectively. These peaks exhibited increased intensity and width. The presence of sawdust and amide B’s CH_2_ stretching vibrations can be identified by two distinct bands observed at 2924.43 and 2855.47 cm^− 1^. The presence of the N–H amide group in sawdust is indicated by a distinct, intense, broad peak at 1604.04 cm^− 1^, corresponding to the amide I band in fish meal. The amide II vibrations of C–N stretching and N–H bending appear at 1507.46 cm^–1^. Figure 7d presents the AC7-800’s FTIR study. The low-intensity peaks spread out and emerge between 3745 and 3096.68 cm^–1^, indicating N–H stretching. Some nitrogen atoms may have hydrogenated, causing these peaks to appear. Broad low-intensity peaks in the C–H stretching may be seen at 2897.11 and 2808.46 cm^− 1^. The low-intensity peaks observed at 2394.36, 2348.46, and 2322.76 cm^− 1^ are caused by the stretching vibrations of the C ≡ N groups in the isonitrile-cyano-terminal. Moderate peaks could be observed between the 2229.50–2048.54 cm^–1^ wavenumbers due to the ketamine (C=C=N) and allene (C=C=C) functional groups. The band exhibits a vibrational frequency of 1705 cm^− 1^ due to the C=C bond. The practical synthesis of nitrogen-doped activated carbon at a temperature of 800 °C was confirmed by distinct and intense peaks characteristic of the sp^2^ structure of the C atoms. The peaks exhibit a significant change between 1508.57 and 1577.73 cm^− 1^ and between 1026.10 and 1107.14 and 1053.24 cm^− 1^. The chaotic structure of the C network may be caused by nitrogen doping, which could be the reason for this movement.

**Fig. 8:**
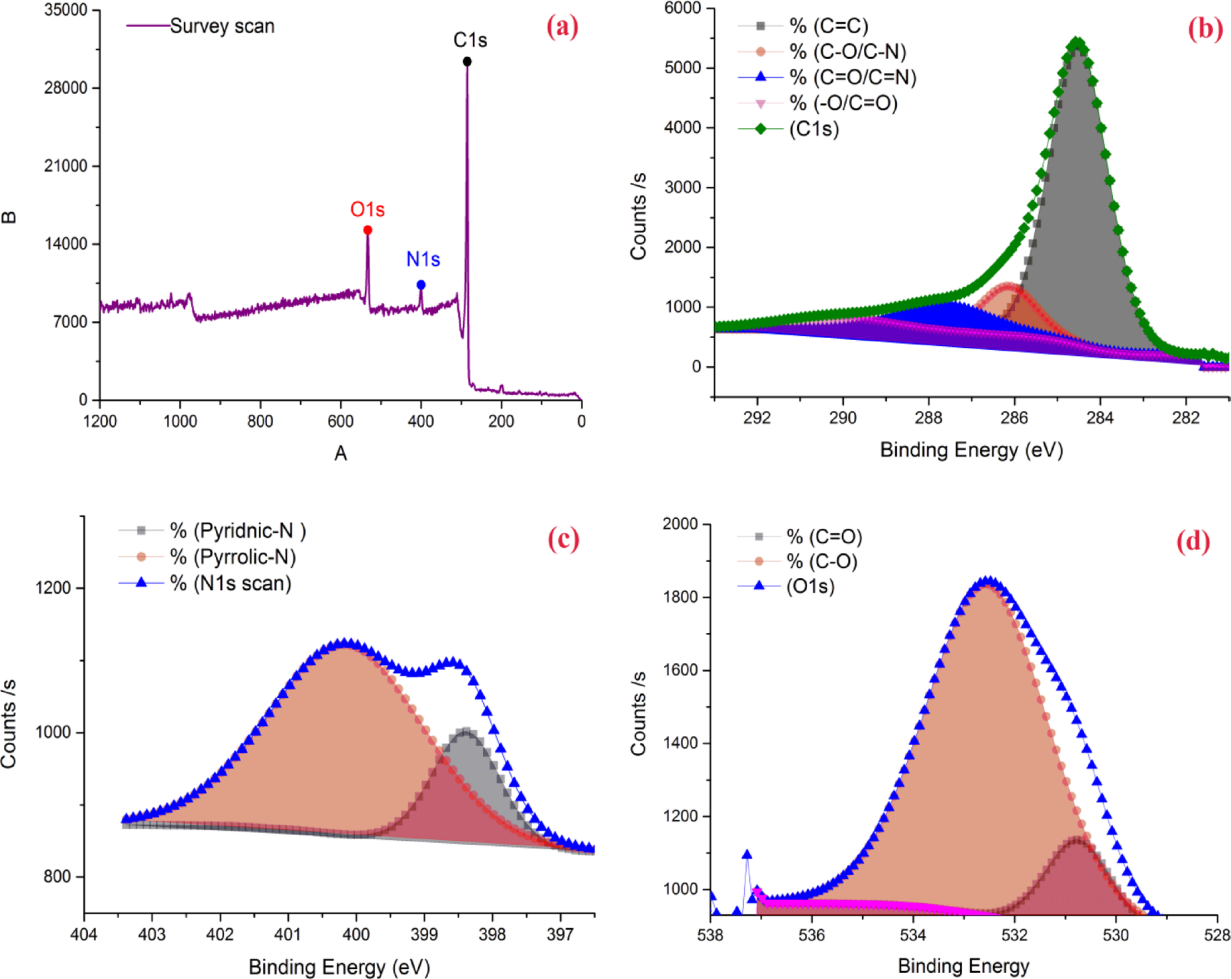
(**a**) AC7-800 survey scan spectrum with 1 eV resolution, (**b**) C1s, (**c**) N1s, and (**d**) O1s high resolution of XPS core level spectra.

X-ray photoelectron spectroscopy (XPS) was used to qualitatively analyze the functional groups on the activated carbon surface^85,86^. The broad complete XPS spectra of the precursor and AC7-800 are illustrated in Fig. 8a. The figures demonstrate that N has been successfully retained on AC7-800, as evidenced by the distinctive peaks of C1s, O1s, and N1s present in AC7-800. The C1s, N1s, and O1s correspond with the peaks at 285.49, 400.33, and 533.05 eV, respectively. Figure 8b, obtained from curve fitting of the C1s spectrum reveals the presence of four distinct peaks in the C1s signal. The C1s spectra display four prominent peaks, located at 284.51 eV (with an intensity of 72.31%), 286.13 eV (with an intensity of 11.15%), 287.68 eV (with an intensity of 9.94%), and 290.18 eV (with an intensity of 6.6%). These peaks are attributed to sp^2^-C hybridized C = C bonds, C–O/C–N bonds, C = O/C=N bonds, and –O/C=O bonds, respectively^87–89^. Two different N-containing chemical types could be identified from the deconvoluted N1s XPS spectra of AC7-800; the outcomes are shown in Fig. 8c. The N1s peak is located in AC7-800 at 400.14 (pyrrolic N) and 398.38 (pyridinic N)^88,90^. Two nitrogen-containing groups are produced from pyrrolic N: 400.14 eV and 398.38 eV (pyridinic N). Pyridinic and pyrrolic N effectively enhance the capacitive properties by promoting the movement of ions from the electrolyte to the electrode material. The O1s XPS spectra of the AC7-800 in Fig. 8d exhibit two distinct peaks at 530.75 eV and 532.54 eV, indicating the presence of (C= O) and (C–O) bonds, respectively.

### Adsorption of AB14 and AY36 Azo dyes on AC7-800

#### Influence of pH

**Fig. 9:**
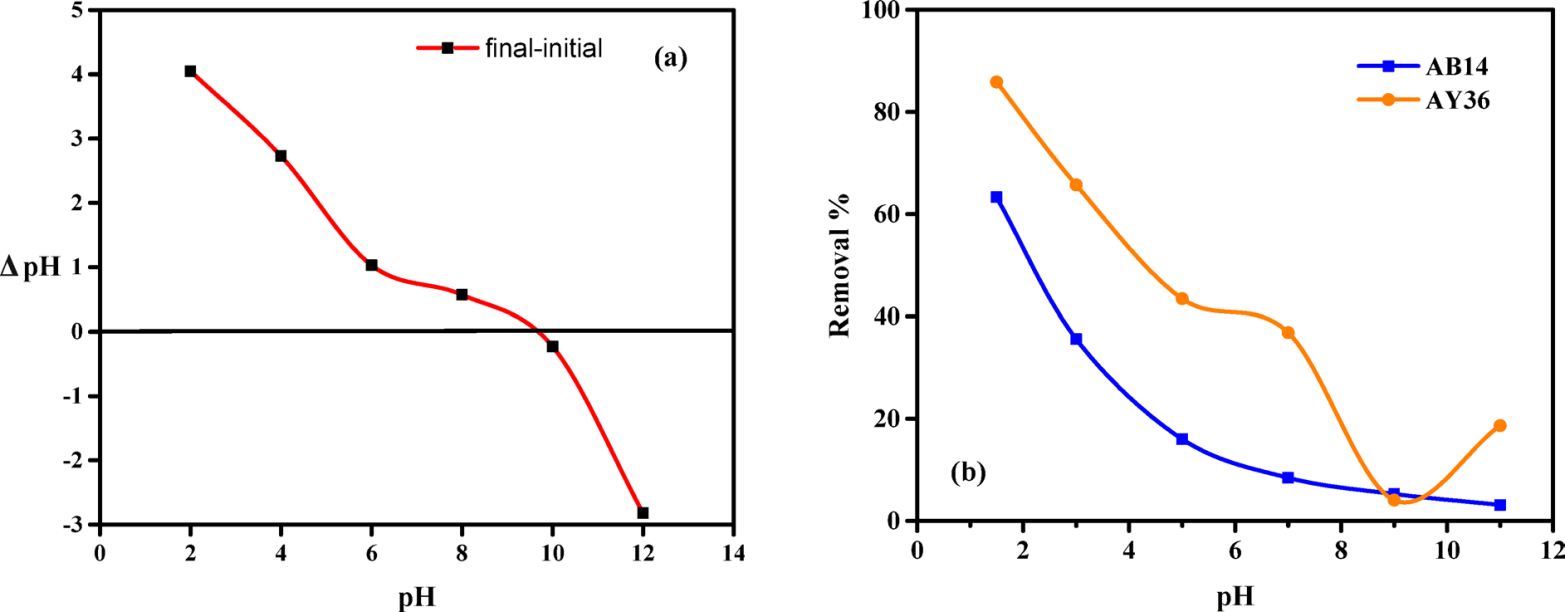
Sorption of AB14 and AY36 dyes onto AC7-800 as a function of (**a**) zero point charge, and (**b**) pH on the removal % of AB14 and AY36 dyes (C_0_ dyes = 100 mg L^–1^, Adsorbent dosage = 0.5 g L^− 1^, Temp. = 25 °C).

The pH of the aqueous solution is crucial in the adsorption of anionic and cationic dyes, as it impacts the ionization of dye molecules and the binding characteristics of the adsorbent surface. To remove AB14 and AY36 dyes, pH values ranging from 1.5 to 11 were investigated. The following conditions were maintained throughout the pH effect studies: 100 mg L^− 1^ dye concentrations at the beginning, 0.5 g L^− 1^ of AC7-800 dosage, 25 °C temperature, and 200 rpm agitation speed. Small amounts of 0.1 M NaOH or HCl were added during the experiment to modify the pH measurements. The pH at which the charge of the point is zero (pH_PZC_) is 9.6, as seen by the curve in Fig. 9a. When the solution’s pH was below the pH_PZC_, the active sites on the biosorbent’s surface exhibited a positive charge.

Conversely, the active sites were negatively charged when the pH was higher than the pH_PZC_. El-Nemr et al.^34^ reported similar findings in their investigation on eliminating Acid Yellow 11 dye. The impact of pH levels on the elimination of AB14 and AY36 dyes from AC7-800 is illustrated in Fig. 9b. The elimination of AB14 dye exhibits a consistent and gradual decline as pH values increase from 1.5 to 11. The efficiency of dye removal in AY36 fell progressively (from 85.86 to 4.07%) as the pH level increased from 1.5 to 9. However, the dye elimination increased to 11.64% when the pH was further elevated to 11. The optimum pH value was 1.5, as they achieved a high removal efficiency of 63.29–85.86% for both dyes. Dye removal efficiencies of AY36 using AC7-800 at all pH values ​​(except pH 9) are higher than those of AB14. At lower pH values, the AB14 and AY36 dye removal efficiency from AC7-800 may be explained by the protonation state of the adsorbent^91,92^. The rise in pH decreased the quantity of positively charged locations and increased the amount of negatively charged locations^93,94^. Consequently, the interaction between the anionic AB14 and AY36 dye molecules and the negatively charged surface of the AC7-800 resulted in electrostatic repulsion.

#### Influence of AC7-800 dosage

**Fig. 10:**
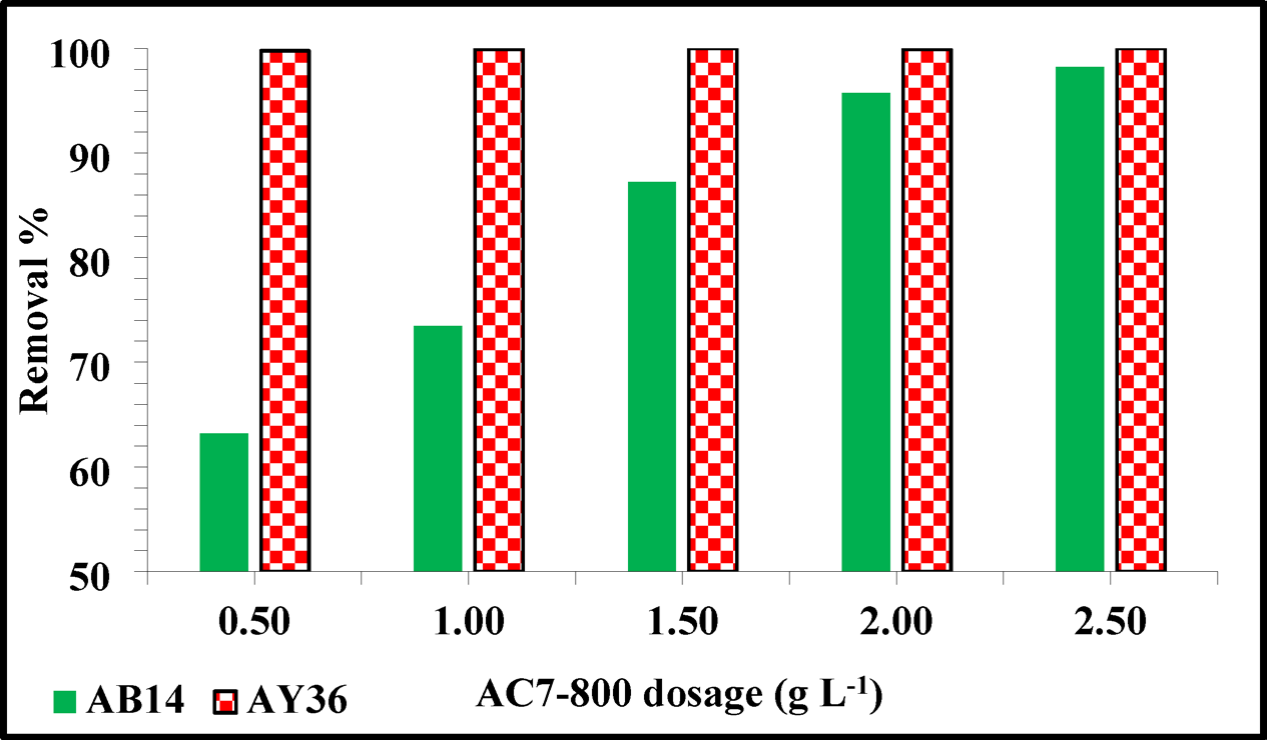
Effect of various AC7-100 dosages on the removal % of AB14 and AY36 dyes.

The adsorbent’s mass effect was examined using various dosages of AC7-800 (0.5, 1.0, 1.5, 2.0, and 2.5 g L^–1^) with beginning concentrations of 100 mg L^–1^ for AB14 and AY36 dye solutions. The duration of contact was 2 h, and the pH levels of the AB14 and AY36 dye solutions were 1.5. To ascertain the final concentrations of the AB14 and AY36 dye solutions, samples were collected at regular intervals (10, 15, 30, 45, 60, 90, and 120 min). Figure 10 illustrates the adsorption of AB14 dye, demonstrating that increasing the bulk of the adsorbent (AC7-100) from 0.5 to 2.5 g L^–1^ resulted in an increment in the elimination percentage from 63.19 to 98.24%. This results from the adsorbent (AC7-100) having unsaturated adsorption sites throughout the adsorption process. Additionally, a large amount of adsorbent can lead to the aggregation of adsorbent particles, resulting in a decrease in adsorption capacity. The adsorbent’s total surface area may decrease due to this aggregation, but the diffusional route length may rise^95^. The change in adsorbent concentration did not have much impact on AY36 dye removal, and removals close to 100% were achieved at almost all concentrations. At all AC7-800 concentrations, AY36 dye removal % is higher than AB14 dye removal %.

#### Influence of initial dye concentrations on AC7-800

**Fig. 11:**
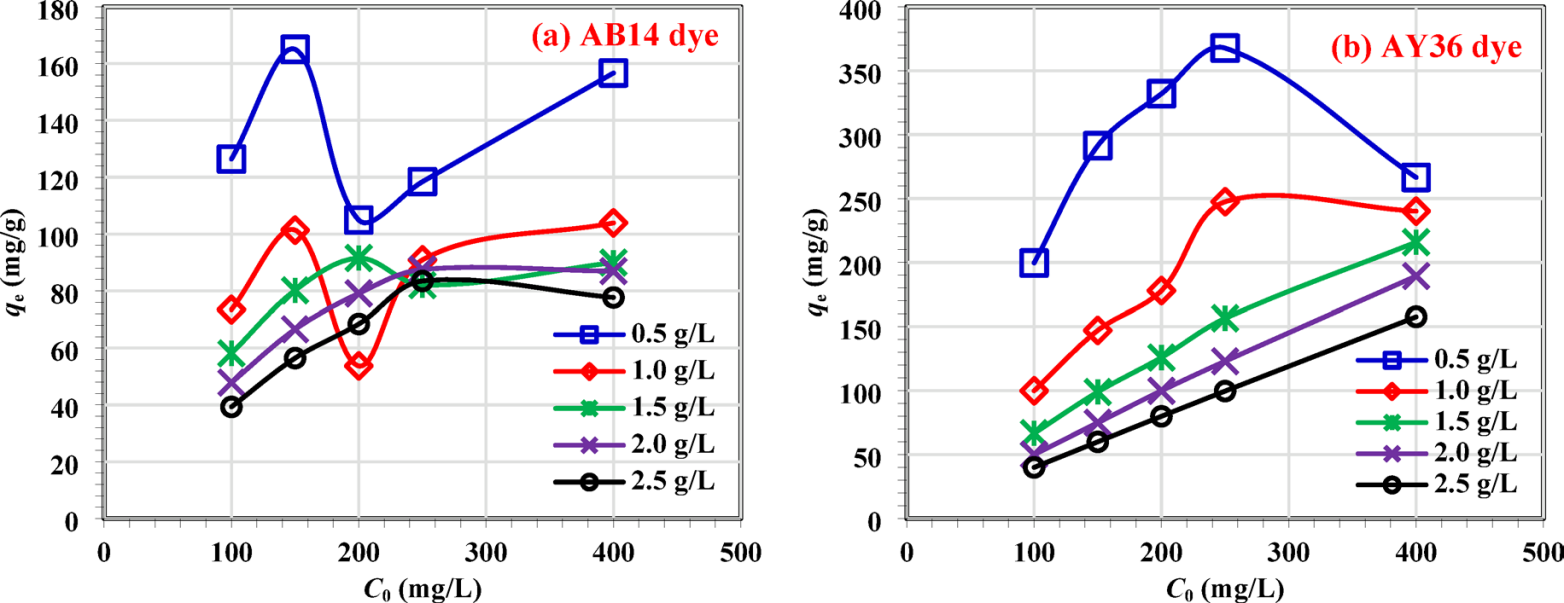
Influence of different concentrations of (**a**) AB14 dye and (**b**) AY36 dye on adsorption capacity *q*_e_ (mg g^–1^) for each AC7-800 concentration at pH of dye solutions = 1.5.

The study investigated the influence of the starting concentrations of AB14 or AY36 dye solutions on the elimination rate. To examine the impact of different starting concentrations (100, 150, 200, 250, and 400 mg L^–1^) and AC7-800 dosages (0.50–2.50 g L^–1^), we experimented with a contact period of 120 min and a pH of 1.5 (already measured). The graph in Fig. 11a shows the relationship between the starting concentration of AB14 dye and the corresponding *Q*_*e*_ (mg g^–1^) values. The *Q*_*e*_ (mg g^–1^) value increased from 126.38 to 156.68 mg g^–1^ when the AC7-800 dosage was 0.50 g L^–1^ and the starting dye concentration ranged from 100 to 400 mg L^–1^. The equilibrium absorption capacity, *Q*_*e*_ (mg g^–1^), rose from 73.46 to 103.91, 58.14 to 89.95, 47.86 to 86.92, and 39.29 to 77.67 mg g^–1^, respectively, when the AC7-800 doses were 1.00, 1.50, 2.00, and 2.50 g L^–1^, and the starting AB14 dye concentration ranged from 100 to 400 mg L^–1^. Upon increasing the dosage of AC7-800 from 0.5 to 2.5 g L^–1^ while maintaining a beginning dye concentration of 400 mg L^–1^, the *Q*_*m*_ of AB14 dye was seen to decline from 156.68 to 77.67 mg g^–1^. In the case of AY36 dye, the value of *Q*_*e*_ (mg g^–1^) increased from 199.58 to 266.45 mg g^–1^ when the AC7-800 dose was 0.50 g L^–1^ and the starting dye concentration ranged from 100 to 400 mg L^–1^ (Fig. 11b). The adsorption capacity at equilibrium, *Q*_*e*_ (mg g^–1^), increased from 99.93 to 240.08, 66.67 to 215.88, 50.00 to 189.32, and 40.00 to 157.68 mg g^–1^, respectively, when the AC7-800 doses were 1.00, 1.50, 2.00, and 2.50 g L^–1^, and the starting AY36 dye concentration ranged from 100 to 400 mg L^–1^. When the dosage of the AC7-800 adsorbent was raised from 0.5 to 2.5 g L^–1^, while the starting dye concentration was kept at 400 mg L^–1^, it was observed that the *Q*_*m*_ of the AY36 dye decreased from 266.46 to 157.68 mg g^–1^. It was observed that AC7-800 adsorbent had higher AY36 dye removal capacities than AB14 dye at all initial concentrations studied.

#### Influence of contact time using AC7-800

**Fig. 12:**
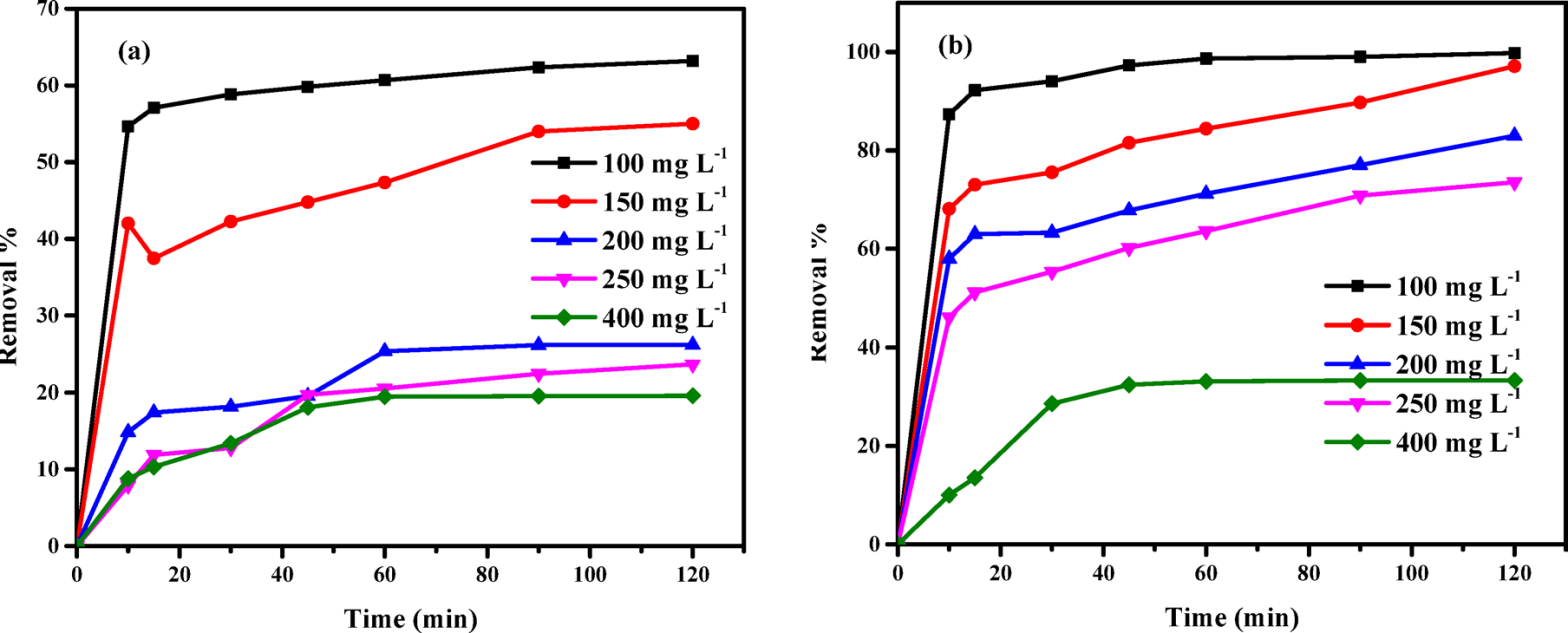
Influence of contact time on adsorption of (**a**) AB14 dye and (**b**) AY36 dye on AC7-800 dose = 0.5 g L^–1^ (pH of dye solutions = 1.5).

Contact time is an inherent aspect of all transfer events, including adsorption. The contact time of the dyes AY36 and AB14 on the removal rates on AC7-800 for 120 min is displayed in Fig. 12. Figure 12a demonstrates that most of the AB14 dye is eliminated during 10 min. The elimination percentage of AB14 dye was 54.65% at a beginning concentration of 100 mg L^–1^ and 8.79% at a beginning concentration of 400 mg L^–1^. Subsequently, there was a gradual and steady rise in the rate of elimination until it reached a state of balance after 120 min, at which juncture it stabilized at 63.19% and 19.58%, correspondingly. If the same comparison is made for the AY36 dye. Figure 12b shows that most AY36 dye is eliminated after 10 min. At 100 mg L^–1^ of the beginning AY36 dye concentration, the elimination percentage was 87.33%, and at 400 mg L^–1^ of the beginning concentration, it was 10.03%. Following that, there was a gradual increase in the elimination rate until it reached equilibrium after 120 min, at this point, it became steady at 99.79 and 33.31%, respectively. The experiment showed that the AC7-800 initially removed increasing amounts of AY36 and AB14 dyes, primarily by exterior surface adsorption. As time progressed, the removal rate steadily decelerated until it reached a state of equilibrium, with internal surface adsorption playing a more significant role. This effect was observed because of the solution’s high concentration and the fact that all of the active sites on the AC7-800 surface were initially vacant. Since there were fewer accessible surface active sites beyond that point, the amount of AY36 and AB14 dyes removed rose very slowly^96^. The AC7-800’s extensive specific surface areas, high pore volumes, porous structures, and adsorbent-adsorbate solid interaction facilitated the material’s rapid uptake of both dyes. The electrostatic attraction observed in AC7-800 is attributed to lone electron pairs on N atoms. This attraction leads to the delocalization of the original sp^2^ hybrid electron cloud on the carbon skeleton, resulting in increased electron transport and surface reactivity.

### Adsorption isotherm studies

The adsorption isotherm is a valuable tool for explaining the mechanisms that govern the retention or movement of substances from water habitats or porous materials to a solid phase under unchanging conditions^97–102^. Various adsorption isotherm models, such as the Langmuir (LIM), the Freundlich (FIM), the Temkin (TIM), and the Halsey isotherm models (HIM), have been formulated to evaluate experimental data^102–104^. As shown in Tables 4 and 5, and Figs. S2 and S3, four adsorption isotherms (LIM, FIM, TIM, and HIM) were examined for the adsorption of both AB14 and AY36 dyes on AC7-800.

#### Langmuir isotherm model (LIM)

The surface has a fixed number of identical sites where adsorption occurs^102–105^. The LIM method was chosen to calculate the *Q*_*m*_ (mg g^–1^) for complete monolayer coverage on the surface of the AC7-800. The expression for LIM is given by Eq. (5).

5 $$\frac{{{C_e}}}{{{q_e}}}=\frac{1}{{{K_L}}}{Q_m}+\frac{1}{{{Q_m}}} \times {C_e}$$

where *Ce* is the concentration of adsorbate in solution at equilibrium (mg L^–1^), *q*_*e*_ is the adsorption capacity at equilibrium (mg g^–1^), *K*_*L*_ is the LIM adsorption constant (L mg^–1^), and *Q*_*m*_ is the maximum adsorption capacity (mg g^–1^).

The LIM data are provided in Tables 4 and 5, and the LIM plots of the dyes AB14 and AY36 are shown in Figs. S2a and S3a, respectively. The AC7-800 adsorbent exhibited strong linear correlations (*R*^2^ = 0.975-1.000) and (*R*^2^ = 0.990–0.998) with the Langmuir model, indicating its effectiveness in removing AB14 and AY36 dyes, respectively. The AB14 and AY36 dyes have monolayer capacities (*Q*_*m*_) of 107.5 and 263.2 mg g^–1^, respectively. The point where the *C*_*e*_*/q*_*e*_ vs. *C*_*e*_ plot intersects and the steepness of the plot are shown in Figs. S2a-S3a were utilized to calculate the values of 1/*Q*_*m*_*K*_*L*_ and 1/*Q*_*m*_ for the LIM, respectively. A strong proof for the dyes’ adsorption on AC7-800 can be found in the *K*_*L*_ ranges of 0.121 to 7.750 L mg^–1^ for AY36 dye and 0.082 to 0.534 L mg^–1^ for AB14 dye, respectively. The LIM states that AY36 and AB14 dyes can be applied to the AC7-800 adsorbent. Upon examination of the AC7-800 adsorbent, it was seen that both dyes were exclusively absorbed in a single layer on its surface.

#### Freundlich isotherm model (FIM)

The FIM was represented as depicted in Eq. (6)^102,106^, :

6 $$Ln~{q_{e=~}}\ln {K_F}+\frac{1}{n}\ln {C_e}$$

where *n* and *K*_*F*_ (mg^1 − 1/n^ g^− 1^ L^1/n^) are the Freundlich constants representing the intensity and capacity of adsorption, respectively.

The FIM data are provided in Tables 4 and 5, and the FIM plots of the AB14 and AY36 dyes are shown in Figs. S2b and S3b, respectively. The point where the ln (*q*_*e*_) vs. ln (*C*_*e*_) plot intersects and the steepness of the plot, as shown in Figures S2b-S3b, respectively, provide the values for log *K*_*F*_ and 1/*n* of the FIM. The binding energy, or *K*_*F*_ (L g^–1^), is one of the FIM constants used to determine how much AY36 or AB14 dye is removed on the AC7-800 at a unit equilibrium concentration. If 1/*n* is smaller than 1, the adsorbent can readily absorb the adsorbate^97^. Therefore, when 1/*n* is less than 1, removing both AY36 and AB14 dyes by AC7-800 adsorbent is a physical process. All values in Tables 4 and 5’s 1/*n* values are less than one, indicating that both dyes can be suitably adsorbed to the AC7-800 adsorbent. The change in log(*q*_*e*_) as a function of log(*C*_*e*_) successfully defines the values of the FIM correlation coefficients (Figs. S2b and S3b). The Freundlich correlation values (*R*^2^ = 0.629–0.999) and (*R*^2^ = 0.478–0.944) for the AC7-800 adsorbent were less than the Langmuir correlation coefficient for the AB14 and AY36 dyes.

#### Temkin isotherm model (TIM)

Equation (7) can be used to define TIM^102,107,108^.

7 $${q_e}=\frac{{RT}}{B}\ln A+\frac{{RT}}{B}ln{C_e}$$

Where *A* and *B* are the TIM constants, *T* is the absolute temperature, and R is the gas constant. A linear plot of *q*_*e*_ versus ln *C*_*e*_ can be used to calculate the constants *A* and *B*, as displayed in Figs. S2c-S3c, in Tables 4 and 5, validated the isotherm’s applicability.

The equilibrium binding constant *A* (g L^–1^) can be calculated by utilizing the slope and intercept of the graph. The graph’s slope calculates the equilibrium binding constant A (g L^–1^), whereas the graph’s intercept is used to estimate the adsorption heat coefficient B. The TIM correlation coefficient for the adsorption of the AB14 and AY36 dyes by the AC7-800 at a dosage of 0.5 g L^–1^ was calculated. Due to the relatively low values of *R*^2^ (0.631–0.994 and 0.427–0.905), it was observed that the model was not suitable for analyzing temperature fluctuations in the elimination process. The AY36 and AB14 dyes were eliminated through physisorption due to the low heat of adsorption and negligible ionic interaction between the adsorbate and adsorbent. The adsorption heat (*B*), determined by the interaction between the adsorbate and adsorbent, does not impact the coating of AY36 and AB14 dyes on the AC7-800 adsorbent. Tables 4 and 5 show that these values fluctuated from 0.5 g L^–1^ AC7-800 to 2.5 g L^–1^.

#### Halsey isotherm model (HIM)

The HIM may be fitted by heteroporous materials and multilayer adsorption^102,109,110^. HIM is represented by Eq. (8):

8 $$ln{q_e}=\frac{1}{n}lnk+\frac{1}{n}ln{C_e}$$

The values of *n* and *k* can be determined from the data obtained from the linear plot of ln *q*_*e*_ vs. ln *C*_*e*_, as illustrated in Figs. S2d-S3d. These values represent the HIM adsorption constants. If this model fits the equilibrium data satisfactorily, the adsorbent is non-homogeneous. According to Figs. S2d-S3d, AB14 dye based on the correction factor is more suitable for fitting such data from TIM at low adsorbent dosages (0.5 and 1.0 g L^–1^) and AY36 dye at all dosages. All in the Halsey model showed a strong association (*R*^2^ = 0.629–0.999) and (*R*^2^ = 0.478–0.944), except for the adsorbent with a concentration of 1.5 g L^–1^ for AB14 dye and 0.5 and 1.0 g L^–1^ for the AY36 dye (Tables 4 and 5), respectively. The multi-layer adsorption within the pores produced elevated R^2^ values when the adsorption data were aligned with the FIM and HIM models.

**Table 4:** Isotherm models data for AB14 dye removal on AC7-800 at room temperature.

Model	(AC7-800) (g L^–1^)				
	0.5	1.0	1.5	2.0	2.5
LIM					
R^2^	0.975	0.993	0.997	1.000	0.999
*Q*_*m*_ (mg g^− 1^)	107.53	105.26	90.91	88.50	78.74
*K*_*L*_	0.082	0.093	0.180	0.251	0.534
FIM					
R^2^	0.999	0.980	0.629	0.888	0.832
*1/n*	0.518	0.138	0.122	0.158	0.155
*K*_*F*_ (mg^1 − 1/n^L^1/n^g^−1^)	7.863	46.366	48.106	40.992	39.364
TIM					
*A*	0.031	16.206	135.915	30.389	100.599
*B*	67.427	11.979	8.918	10.570	8.097
R^2^	0.994	0.965	0.631	0.909	0.961
HIM					
*1/n*_*H*_	0.518	0.138	0.122	0.158	0.155
*k*	53.657	1.14 × 10^12^	5.99 × 10^13^	1.56 × 10^10^	2.05 × 10^10^
R^2^	0.999	0.980	0.629	0.888	0.832

**Table 5:** Adsorption isotherm data for AY36 dye removal on AC7-800 at room temperature.

Model	(AC7-800) (g L^− 1^)				
	0.5	1.0	1.5	2.0	2.5
LIM					
R^2^	0.996	0.998	0.992	0.990	0.994
*Q*_*m*_ (mg g^− 1^)	263.16	243.90	232.56	192.31	161.29
*K*_*L*_	0.121	0.320	0.243	1.793	7.750
FIM					
R^2^	0.478	0.606	0.944	0.868	0.939
*1/n*	0.058	0.102	0.241	0.145	0.152
*K*_*F*_ (mg^1 − 1/n^L^1/n^g^−1^)	244.51	148.70	87.90	112.88	114.45
TIM					
*A*	1.7 × 10^7^	2.6 × 10^4^	13.58	720.20	704.13
*B*	15.104	15.707	29.451	18.170	17.296
R^2^	0.427	0.523	0.905	0.863	0.897
HIM					
*1/n*_*H*_	0.058	0.102	0.241	0.149	0.112
*k*	7.88 × 10^40^	2.4 × 10^21^	6.43 × 10^7^	1.99 × 10^13^	9.96 × 10^16^
R^2^	0.478	0.606	0.944	0.868	0.939

#### Error function examination for best-fit isotherm models

The correlation coefficients (*R*^2^) of the LIM, FIM, TIM, and HIM models^92^ were compared with experimental equilibrium data to identify the most appropriate model for the adsorption of either AB14 or AY36 dyes onto the AC7-800 adsorbent. Another method of determining the best model is to compare the error functions. These can be listed as the Average percent errors (APE), Chi-square error (X^2^), hybrid error function (HYBRID), the Sum Square Error (ERRSQ), Marquardt’s percent standard deviation (MPSD), Average relative error (ARE), Sum of absolute errors (EABS), and root mean square errors (RMS)^111^. Upon comparing all error functions (Tables 6 and 7), it can be concluded that the TIM effectively matches the experimental data. This is evident from the models that exhibit values closest to zero, indicating their suitability as the most suited models.

**Table 6:** Error function analysis values of the isotherm models used to remove AB14 dye on AC7-800.

Model	X^2^	APE (%)	Hybrid	MPSD	ERRSQ	ARE	RMS	EABS
LIM	9.452	0.276	41.098	1.441	748.463	0.276	1.382	136.790
FIM	0.008	0.008	0.034	0.044	0.548	0.008	0.042	3.700
TIM	7.3 × 10^− 10^	2.6 × 10^− 6^	3.2 × 10^− 9^	1.4 × 10^− 5^	4.8 × 10^− 8^	2.6 × 10^− 6^	1.3 × 10^− 5^	1.1 × 10^− 3^
HIM	0.008	0.009	0.034	0.044	0.550	0.009	0.043	3.707

**Table 7:** Error function analysis values of the isotherm models used to remove AY36 dye on AC7-800.

Model	X^2^	APE (%)	Hybrid	MPSD	ERRSQ	ARE	RMS	EABS
LIM	159.050	0.835	691.523	4.351	23240.3	0.835	4.173	762.239
FIM	0.358	0.039	1.556	0.203	54.267	0.039	0.194	36.833
TIM	0.050	0.014	0.216	0.075	7.534	0.014	0.072	13.724
HIM	12.903	0.233	56.099	1.216	1956.829	0.233	1.167	221.180

### Adsorption kinetic studies

An analysis of many models, including diffusion control, mass transfer, and chemical reaction, was conducted to study the kinetics of adsorption. The study aimed to examine the kinetics of the adsorption of AB14 and AY36 dyes onto AC7-800. The aim was to determine the optimal working conditions for a full-scale batch process. Comprehending the kinetic parameters is vital for forecasting the pace at which adsorption occurs and supplying necessary proof for developing and simulating adsorption processes. Therefore, the kinetic models^102^ of pseudo-first order (PFOM)^112^, pseudo-second-order (PSOM)^113^, Elovich (EM)^114–116^, intraparticle diffusion (IDM), and film diffusion (FDM)^117,118^ were employed to study the removal of either AB14 or AY36 dye onto AC7-800. The results are presented in Tables 8, 9, 10 and 11. The level of concordance between the values predicted by the model and the actual experimental data was measured using the *R*^2^ (coefficient of determination) displayed in Figs. S4-S5 and Tables 8, 9, 10 and 11.

#### Pseudofirstorder model (PFOM)

The pseudo-first-order (PFO) kinetic model is represented by Eq. (9)^119^, .

9 $$\log \left( {{q_e} - {q_t}} \right)=\log \left( {{q_e}} \right) - \frac{{{k_1}}}{{2.303}}t$$

Where *q*_*t*_ and *q*_*e*_ (mg g^–1^) represent the amounts of ion adsorbed at time *t* and equilibrium, respectively. *k*_1_ (min^− 1^) is the rate constant of the PFO adsorption process. The values of *k*_1_ and *q*_*e*_ were determined by calculating the slope and intercept of the plots of log (*q*_*e*_ − *q*_*t*_) vs. *t*, as shown in Figs. S4a-S5a. The kinetic models in Tables 8 and 10 demonstrate correlation values (*R*^2^) ranging from 0 to 1. The model’s appropriateness is directly proportional to the proximity of the *R*^2^ value to one. The *R*^2^ values, mostly above 0.900 with a few exceptions, indicate a strong correlation between the estimated *q*_*e*_ values and the experimental *q*_*e*_ values. Based on the data shown in Tables 8 and 10, it can be concluded that the PFOM equation is suitable for modelling the adsorption of AB14 and AY36 dyes on AC7-800. Tables 8 and 10 demonstrate no consistent pattern of increase or decrease in *R*^2^ values when the concentration of the AC7-800 increases from 0.5 to 2.5 g L^–1^.

#### Pseudosecondorder model (PSOM)

The *q*_*e*_–*q*_*t*_ is directly proportional to the number of active sites on the adsorbent^120,121^. The PSOM can be calculated by Eq. (10):

10 $$\left( {\frac{t}{{{q_t}}}} \right)=\frac{1}{{{k_2}q_{e}^{2}}}+\frac{1}{{{q_e}}}\left( t \right)$$

where *k*_*2*_ (g mg^− 1^ min^− 1^) is the equilibrium rate constant for PSOM adsorption^102,122^. If the PSOM is suitable, the plot of *t*/*q*_*t*_ vs. *t* exhibits a linear correlation. The values of *k*_*2*_ and *q*_*e*_ can be determined from the slope and intercept of the line, respectively. These relationships are illustrated in Figs. S4b and S5b depict the PSOM curve of the AC7-800 for the elimination of the AB14 and AY36 dyes, respectively. Tables 8 and 10 contain the PSOM constant (*k*^2^) values, the theoretical and experimentally obtained *q*_e_ values, and the corresponding *R*^2^ values. Tables 8 and 10 indicate that the PSOM has *R*^2^ values close to 1. The PSOM is the most appropriate kinetic model for both dyes. The experimental *q*_*e*_ values precisely coincide with the estimated *q*_*e*_ values for all the tested initial concentrations of AB14 and AY36 dyes.

#### Elovich model (EM)

EM is used to investigate various active sites on the adsorbent material^123,124^. Equation (11) represents the presentation of EM.

11 $${q_t}=\frac{1}{\beta }\ln \left( {\alpha \beta } \right)+\frac{1}{\beta }{\text{ln}}\left( t \right)$$

Where *α* (mg g^–1^ min^–1^) and *β* (g mg^–1^) denote the initial sorption rate constant and the surface coverage and activation energy for chemisorption, respectively. The values of these constants can be determined by calculating the slope and intercept of the plot of *q*_*t*_ versus ln *t*, using the model equation (Figs. S4c-S5c). The EM constants were determined by calculating the intercept and slope of Figs. S4c and S5c, respectively. The computed values are presented in Tables 9 and 11. When comparing the *R*^2^ values, it can be observed that the *R*^2^ values of EM are marginally greater than those of PFOM and less than those of PSOM (Tables 8, 10 and 11). The findings from Tables 9 and 11 indicate that chemical adsorption can regulate the rate at which AB14 and AY36 dyes are adsorbed on the AC7-800 adsorbent, but only in specific situations.

#### Intraparticle diffusion model (IPDM)

The isothermal plug flow diffusion (IPD) may be the rate-controlling step in an experimental setup employing a batch technique and vigorous agitation^102,125,126^. The investigation of the IPDM also involved the use of Eq. (12)^127^:

12 $${q_t}={K_{diff}}{t^{0.5}}+C$$

where *K*_*diff*_ represents the rate constant of IPD (mg g^− 1^ min^1/2^) (Figs. S4d and S5d).

The hypothesis proposed by Weber and Morris^102,114^ suggests that intraparticle diffusion step controls the adsorption process if the lines depicted in the graph of *q*_*t*_ and the square root of time (*t*) in Figs. S4d and S5d intersect at the origin. Nevertheless, if the lines do not pass through the origin, it is believed that FD controls the rate of the elimination process, particularly as the *C* value is large. The removal of either AB14 or AY36 dyes onto AC7-800 was examined at various adsorbent doses and beginning dye concentrations. The Webber-Morris adsorption lines for AB14 and AY36 dyes are depicted in Figs. S4d and S5d, respectively. The *K*_*dif*_ and *C* values shown in Tables 9 and 11 were derived from the slope and intercept points of the plot of *q*_*t*_ vs. *t*^0.5^. The straight lines in Figs. S4d and S5d, which show all adsorbent concentrations, do not intersect at the origin of both dyes due to their high *C* intersection. This can be proven by the fact that FD controls the speed at which both dyes are absorbed into the AC7-800 adsorbent. This absorption rate gradually rises over time, as seen in Figs. S4e-S5e. This phenomenon arises due to the gradual reduction in the surface area and pore volume of the AC7-800 adsorbent during removal.

#### Film diffusion model (FDM)

Film diffusion (FD) occurs when the adsorbate molecules move across the liquid film around the adsorbent particle. Equation (13) gives the equation for FDM.

13 $$\ln \left( {1 - F} \right)={K_{FD}}\left( t \right)$$

*K*_*FD*_ represents the external film mass transfer coefficient, and *F* is the ratio of *q*_*t*_ to *q*_*e*_. The constant *K*_*FD*_ can be determined by calculating the slope and intercept of the ln (1 − *F*) against the plot (Figs. S4e and S5e)^117^.

The PSOM was determined to be the best-fit kinetic model for removing AB14 and AY36 dyes by AC7-800. This conclusion was based on the highest determination coefficient (*R*^2^ = 1) obtained from the PSOM rate data. The electrostatic attraction between the negatively charged active sites on the self-doping activated carbon and the hydrogen ions in the solution is thought to be the initial stage of the process, based on the PSOM, the LIM, and the TIM adsorption isotherm models. This compound contained several nitrogen atoms that possessed unshared pairs of electrons. Both dyes were then taken up by the surface of the activated carbon, which had been positively self-doped, creating a noticeable adsorption layer.

**Table 8:** Comparing the estimated and experimental *q*_e_ values for different starting AB14 dye and AC7-800 concentrations and the PFOM and PSOM adsorption rate constants.

Parameter			PFOM			PSOM		
AC7-800 (g L^− 1^)	AB14 dye (mg L^− 1^)	*q*_*e*_ (exp.)	*q*_*e*_ (calc.)	*k*_*1*_ × 10^3^	R^2^	*q*_*e*_ (calc.)	*k*_*2*_ × 10^3^	R^2^
0.5	100	199.58	20.76	35.70	0.977	128.21	3.826	1.000
	150	291.32	86.32	31.32	0.819	175.44	0.607	0.992
	200	331.94	182.31	72.08	0.869	117.65	0.627	0.992
	250	367.66	107.15	32.24	0.968	144.93	0.258	0.981
	400	266.46	247.51	76.23	0.935	181.82	0.347	0.990
1.0	100	99.93	6.22	20.50	0.674	74.07	9.205	1.000
	150	147.13	11.03	23.26	0.920	55.25	6.513	1.000
	200	178.01	15.24	46.36	0.905	102.04	5.584	1.000
	250	247.48	34.45	13.59	0.860	93.46	1.080	0.989
	400	240.08	78.25	22.80	0.894	119.05	0.348	0.950
1.5	100	66.67	18.98	62.64	0.783	58.82	8.611	1.000
	150	99.02	8.23	16.12	0.949	80.65	6.417	1.000
	200	125.96	95.02	40.30	0.935	106.38	0.477	0.992
	250	156.49	42.16	21.65	0.880	86.96	0.951	0.986
	400	215.88	26.27	33.16	0.909	92.59	2.541	1.000
2.0	100	50.00	5.34	26.71	0.823	48.31	12.753	1.000
	150	74.93	10.37	26.95	0.988	67.57	6.500	1.000
	200	99.93	32.55	41.91	0.893	82.64	2.324	0.999
	250	123.04	40.49	19.58	0.851	93.46	0.892	0.984
	400	189.32	38.73	22.11	0.979	91.74	1.140	0.995
2.5	100	40.00	12.66	42.84	0.887	39.84	13.908	1.000
	150	60.00	13.72	21.88	0.955	57.47	7.783	1.000
	200	79.97	32.61	34.08	0.888	69.44	4.164	0.999
	250	99.69	6.01	36.16	0.956	87.72	1.785	0.999
	400	157.68	25.37	10.13	0.901	82.64	1.171	0.985

**Table 9:** Comparison of the EM, IPDM, and FDM adsorption rate constants for various initial AB14 dye and AC7-800 concentrations.

Parameter		EM			IPDM			FDM		
AC7-800 (g L^− 1^)	AB14 dye (mg L^− 1^)	*β*	α	R^2^	*K*_*dif*_	*C*	R^2^	K_FD_	*C*	R^2^
0.5	100	0.15	1.65 × 10^7^	0.986	2.03	105.3	0.951	0.03	1.81	0.977
	150	0.05	697.0	0.804	6.42	94.84	0.893	0.03	0.65	0.819
	200	0.05	37.9	0.890	6.26	42.05	0.893	0.07	0.55	0.869
	250	0.03	11.5	0.947	10.14	16.27	0.909	0.03	0.10	0.968
	400	0.03	24.0	0.926	12.05	43.15	0.844	0.08	0.46	0.935
1.0	100	0.41	2 × 10^11^	0.843	0.76	65.47	0.794	0.02	2.47	0.674
	150	0.30	2.41 × 10^11^	0.900	1.09	89.70	0.905	0.01	0.97	0.860
	200	0.21	4130	0.856	1.42	40.57	0.722	0.02	2.22	0.920
	250	0.08	104	0.884	3.99	45.68	0.868	0.02	0.28	0.894
	400	0.05	18.3	0.832	6.99	25.82	0.927	0.05	1.26	0.905
1.5	100	0.46	6.04 × 10^9^	0.968	0.71	50.77	0.928	0.06	1.12	0.783
	150	0.37	1.66 × 10^11^	0.932	0.87	70.39	0.966	0.02	2.28	0.949
	200	0.05	13.9	0.940	6.74	22.88	0.972	0.04	0.04	0.935
	250	0.08	58.9	0.968	4.04	37.47	0.953	0.02	0.67	0.880
	400	0.10	707	0.978	3.07	60.39	0.794	0.03	1.23	0.909
2.0	100	0.46	6.84 × 10^7^	0.892	0.66	41.32	0.791	0.03	2.19	0.823
	150	0.31	2.47 × 10^7^	0.994	1.02	56.07	0.963	0.03	1.86	0.988
	200	0.10	332	0.932	2.98	50.39	0.836	0.04	0.89	0.893
	250	0.08	90.1	0.816	4.07	42.24	0.863	0.02	0.77	0.851
	400	0.08	113	0.986	3.89	45.08	0.991	0.02	0.81	0.979
2.5	100	0.68	4.41 × 10^9^	0.941	0.47	34.45	0.947	0.04	1.88	0.956
	150	0.38	6.05 × 10^7^	0.963	0.83	48.02	0.950	0.02	2.06	0.954
	200	1.60	5.09 × 10^31^	0.966	1.46	52.94	0.943	0.02	1.61	0.955
	250	0.08	93.7	0.900	3.85	46.62	0.785	0.03	0.94	0.888
	400	0.10	203	0.820	3.22	42.56	0.902	0.01	1.14	0.948

**Table 10:** Comparing the estimated and experimental *q*_e_ values for different starting AY36 dye and AC7-800 concentrations and the PFOM and PSOM adsorption rate constants.

Parameter			PFOM			PSOM		
AC7-800 (g L^− 1^)	AY36 dye (mg L^− 1^)	*q*_*e*_ (exp.)	*q*_*e*_ (calc.)	*k*_*1*_ × 10^3^	R^2^	*q*_*e*_ (calc.)	*k*_*2*_ × 10^3^	R^2^
0.5	100	199.58	28.72	35.70	0.943	204.08	2.82	1.000
	150	291.32	99.98	*16.58*	0.991	303.03	0.41	0.994
	200	331.94	119.07	16.81	0.961	344.83	0.34	0.992
	250	367.66	194.54	26.95	0.951	400.00	0.24	0.995
	400	266.46	310.10	72.77	0.915	333.33	0.13	0.958
1.0	100	99.93	7.19	28.79	0.673	101.01	9.08	1.000
	150	147.13	15.88	20.04	0.932	149.25	3.62	1.000
	200	178.01	36.72	40.30	0.897	181.82	2.52	1.000
	250	247.48	115.85	36.39	0.877	256.41	0.61	0.997
	400	240.08	641.06	70.70	0.787	303.03	0.11	0.942
1.5	100	66.67	1.58	28.10	0.977	66.67	48.91	1.000
	150	99.02	2.89	19.35	0.849	99.01	22.18	1.000
	200	125.96	25.62	38.46	0.918	128.21	3.22	1.000
	250	156.49	45.46	45.14	0.912	158.73	2.24	1.000
	400	215.88	352.05	73.01	0.826	232.56	0.43	0.997
2.0	100	50.00	1.87	40.99	0.904	50.00	181.82	1.000
	150	74.93	1.89	34.55	0.978	75.19	52.03	1.000
	200	99.93	3.26	21.88	0.477	100.00	18.18	1.000
	250	123.04	3.55	13.82	0.980	123.46	13.67	1.000
	400	189.32	33.87	47.90	0.914	192.31	3.07	1.000
2.5	100	40.00	0.01	-52.51	0.735	40.00	1562.50	1.000
	150	60.00	0.03	-30.63	0.618	59.88	557.78	1.000
	200	79.97	0.90	10.13	0.124	80.00	52.08	1.000
	250	99.69	3.79	25.56	0.974	100.00	18.18	1.000
	400	157.68	14.03	57.81	0.933	158.73	9.92	1.000

**Table 11:** Comparison of the EM, IPDM, and FDM adsorption rate constants for various initial AY36 dye and AC7-800 concentrations.

Parameter		EM			IPDM			FDM		
AC7-800 (g L^− 1^)	AY36 dye (mg L^− 1^)	*β*	α	R^2^	*K*_*dif*_	*C*	R^2^	K_FD_	*C*	R^2^
0.5	100	0.10	1.08 × 10^8^	0.933	2.92	171.21	0.842	0.04	1.94	0.943
	150	0.03	1650	0.946	10.49	173,03	0.987	0.02	1.07	0.991
	200	0.03	1860	0.905	12.04	194,58	0.969	0.02	1.03	0.961
	250	0.02	351	0.977	17.51	181,92	0.989	0.03	0.64	0.951
	400	0.01	27.6	0.855	24.10	47,40	0.721	0.01	1.40	0.036
1.0	100	0.37	4.14 × 10^14^	0.908	0.85	91,67	0.884	-0.02	3.91	0.114
	150	0.19	2.88 × 10^10^	0.949	1.72	128,61	0.957	0.02	2.23	0.932
	200	0.10	1.02 × 10^7^	0.878	2.93	148,99	0.797	0.04	1.58	0.897
	250	0.04	2330	0.969	8.51	158,81	0.969	0.04	0.76	0.877
	400	0.02	26.8	0.872	19.58	37,84	0.920	0.07	-0.98	0.787
1.5	100	1.85	2.26 × 10^51^	0.968	0.17	64,98	0.906	0.03	3.74	0.977
	150	0.85	2.88 × 10^34^	0.932	0.36	95,23	0.851	0.02	3.53	0.849
	200	0.13	8.66 × 10^5^	0.940	2.39	102,81	0.866	-0.01	2.94	0.073
	250	0.11	3.21 × 10^6^	0.968	2.85	127.55	0.966	0.05	1,24	0.912
	400	0.03	136	0.978	11.35	102.71	0.949	0.07	-0,49	0.826
2.0	100	6.67	6.54 × 10^141^	0.277	0.04	49.52	0.230	0.07	0,60	0.544
	150	2.20	1.39 × 10^69^	0.944	0.14	73.47	0.933	0.03	3,79	0.978
	200	0.68	3.78 × 10^27^	0.958	0.46	95.45	0.913	-0.03	4,70	0.169
	250	0.75	2.08 × 10^38^	0.899	0.43	118.41	0.944	-0.04	4,88	0.471
	400	0.09	7.65 × 10^6^	0.877	3.13	159.90	0.755	0.05	1,72	0.914
2.5	100	74.07		0.677	0.00	39.95	0.738	-0.11	9,29	0.735
	150	45.25		0.545	0.01	59.91	0.680	-0.07	8,91	0.618
	200	1.60	2.79 × 10^53^	0.947	0.19	78.18	0.844	-0.04	5,70	0.251
	250	0.92	7.98 × 10^37^	0.959	0.35	95.95	0.988	0.03	3,27	0.974
	400	0.30	1.88 × 10^19^	0.772	1.00	148.28	0.687	0.06	2,42	0.933

### Comparison of results with literature

The literature review compared the effectiveness of several adsorbents in removing AB14 and AY36 dyes with the AC7-800 adsorbent. Table 12 compares the highest adsorption capacities (measured in milligrams per gram) of the adsorbents utilized in this investigation with other findings documented in existing literature. Table 12 demonstrates that the AC7-800 adsorbent effectively removes AB14 and AY36 dyes.

**Table 12:** A comparison of the most effective adsorbents for removing pollutants.

Adsorbent	Dye	Conditions	Q_m_ (mg·g^− 1^)	References
Nitrogen-doped activated carbon (NDAC)	AB14	pH 1.5, 100 ppm, 1.0 g/L, removal 89.03%	909.09	^75^
Magnetic carbon nano-composite	AB14	pH 4.6, 20 ppm, 0.8 g /L, removal 82%	20.50	^128^
Double hydroxide Calcined Mg/Fe layered	AB14	pH 4, 40 ppm, 0.2 g /L, removal 100%	370.00	^129^
Mandarin‑CO‑TETA (MCT)	AB14	pH 1.5, 100 ppm, 1.0 g/L, removal 86.9%	416.67	^12^
Sawdust carbon (SDC)	AY36	pH 3, 1000 ppm, 0.2 g/L, removal ~ 100%	183.80	^130^
Rice husk carbon (RHC)	AY36	pH 3, 1000 ppm, 0.8 g/L, removal ~ 80%	86.9	^130^
Green nanoceria (GN)	AY36	pH 2, 10 ppm, 0.05 g/L, removal ~ 73.3%	16.39	^131^
Amine functionalized to GN-NH_2_ (AGN)	AY36	pH 2, 10 ppm, 0.05 g/L, removal ~ 92.9%	26.95	^131^
AC7-800	AB14	pH 1.5, 100 ppm, 0.5 g/L, removal 54.65%	107.53	This Study
AC7-800	AY36	pH 1.5, 100 ppm, 0.5 g/L, removal 87.33%	263.16	This Study

(AB14:acid brown 14, AY36:acid yellow 36)

### RSM optimization study

A design matrix was utilized to study the interactive impacts of contact time, catalyst dosage, and initial dye concentration on removing AB14 and AY36 dyes. Table S1 displays the experimental design and the associated responses. Polynomial equations were later formulated using the acquired data to characterize the removal effectiveness of AB14 dye (Eqs. 14,15):

14 $$\begin{aligned} {\text{Removal }}\,\% \,{\text{ for}}\,{\text{ Coded }}\,{\text{Factors}}\,= & {\text{44}}.{\text{21}}\,+\,{\text{17}}.{\text{82A}} - {\text{24}}.{\text{61B}}\,+\,{\text{3}}.{\text{93C}} - {\text{1}}.{\text{94AB}} - 0.{\text{5826AC}} \\ \quad & - 0.{\text{3466BC}}--{\text{ 3}}.{\text{78}}{{\text{A}}^{\text{2}}}\,+\,0.{\text{11}}.{\text{9}}0{{\text{B}}^{\text{2}}}\,+\,{\text{1}}.{\text{36}}{{\text{C}}^{\text{2}}} \\ \end{aligned}$$

15 $$\begin{aligned} {\text{Removal}}\,{\text{ }}\% {\text{ }}\,{\text{for}}\,{\text{ Actual}}\,{\text{ Factors}}\, = & {\text{73}}.{\text{5}}\, + \,0.{\text{331AC7}} - {\text{8}}00{\text{ dosage}} - \,0.{\text{4}}0{\text{6Dye Conc}}. \\ \quad & + 0.0{\text{358Time}}. - 0.0000{\text{1AC7}} - {\text{8}}00{\text{ dosage}} \times {\text{Dye Conc}}. - 0.000{\text{1AC7}} \\ \quad & - {\text{8}}00{\text{ dosage}} \times {\text{Time}} - 0.0000{\text{4 Dye Conc}}. \times {\text{Time}}{-}0.000{\text{378 AC7}} \\ \quad & - {\text{8}}00{\text{ dosage}}^{{\text{2}}} \, + \,0.0000{\text{529 Dye Conc}}.^{{\text{2}}} + 0.000{\text{493 Time}}^{{\text{2}}} \\ \end{aligned}$$

The subsequent polynomial equations for the elimination of AY36 were formulated (Eqs. 16,17):

16 $$\begin{aligned} {\text{Removal}}\,{\text{ }}\% \,{\text{ for }}\,{\text{Coded}}\,{\text{ Factors}}= & {\text{94}}.{\text{18}}\,+\,{\text{19}}.{\text{56A}} - {\text{16}}.{\text{39B}}\,+\,{\text{4}}.{\text{23C}} \\ \quad & +{\text{17}}.{\text{47AB}} - {\text{4}}.{\text{31AC}}\,+\,{\text{1}}.0{\text{BC}}-{\text{ 11}}.0{\text{7}}{{\text{A}}^{\text{2}}} - {\text{2}}.{\text{41}}{{\text{B}}^{\text{2}}} - {\text{2}}.{\text{9}}0{{\text{C}}^{\text{2}}} \\ \end{aligned}$$

17 $$\begin{aligned} {\text{Removal }}\% {\text{ for Actual Factors}}\, = & {\text{87}}.{\text{8}}\, + \,0.{\text{291 AC7}} - {\text{8}}00{\text{ dosage}} - \,0.{\text{239 Dye Conc}}.0.{\text{314Time}}. \\ \quad & + 0.00{\text{11 AC7}} - {\text{8}}00{\text{ dosage}} \times {\text{Dye Conc}}. \\ \quad & - 0.000{\text{8 AC7}} - {\text{8}}00{\text{ dosage}} \times {\text{Time}} + 0.000{\text{12 Dye Conc}}. \times {\text{Time}} \\ \quad & {-}0.00{\text{11 AC7}} - {\text{8}}00^{{\text{2}}} - 0.000{\text{1 Dye Conc}}.^{{\text{2}}} - \,0.00{\text{1}}0{\text{ Time}}^{{\text{2}}} \\ \end{aligned}$$

The reaction may be predicted using the equation stated in actual factors at specific values of each component, which are given in their original units. Since the coefficients are modified to match the units of each element and the intercept is not located in the centre of the design space, this equation should not be used to determine the relative importance of each factor.

Figure S6a, b illustrates the association between the expected and actual adsorption percentages of AB14 and AY36 dyes on AC7-800. Figure S6 demonstrates a strong concordance between the experimental findings and the anticipated model, as evidenced by the elevated correlation coefficients (R^2^ = 0.992 and 0.993) for AB14 and AY36 dyes, respectively. The ANOVA presented in Tables S2 and S3 is utilized to forecast the independent factors’ cubic, individual, and interaction effects on the adsorption of AB14 and AY36 dyes on AC7-800. The findings indicate that the quadratic model (P-value < 0.0001) makes a substantial contribution. The coefficient of determination indicates the quality of the polynomial model based on the degree of divergence from the mean represented by the model, and the values of Adj-R2 demonstrate a strong correlation between the expected and observed data^132,133^.

The predicted *R*^2^ aligns reasonably with the Adjusted R^2^ of AB14 and AY36 dyes, with a difference of less than 0.2. Adeq Precision quantifies the signal-to-noise (S/N) ratio. A ratio over 4 is preferable. The S/N values of 40.06 and 34.90 for AB14 and AY36 dyes, respectively, signify a satisfactory signal, demonstrating a substantial RSM model signal that can be employed for guiding the design^132,134^.

#### Simultaneous effects of interactive adsorption variables

**Fig. 13:**
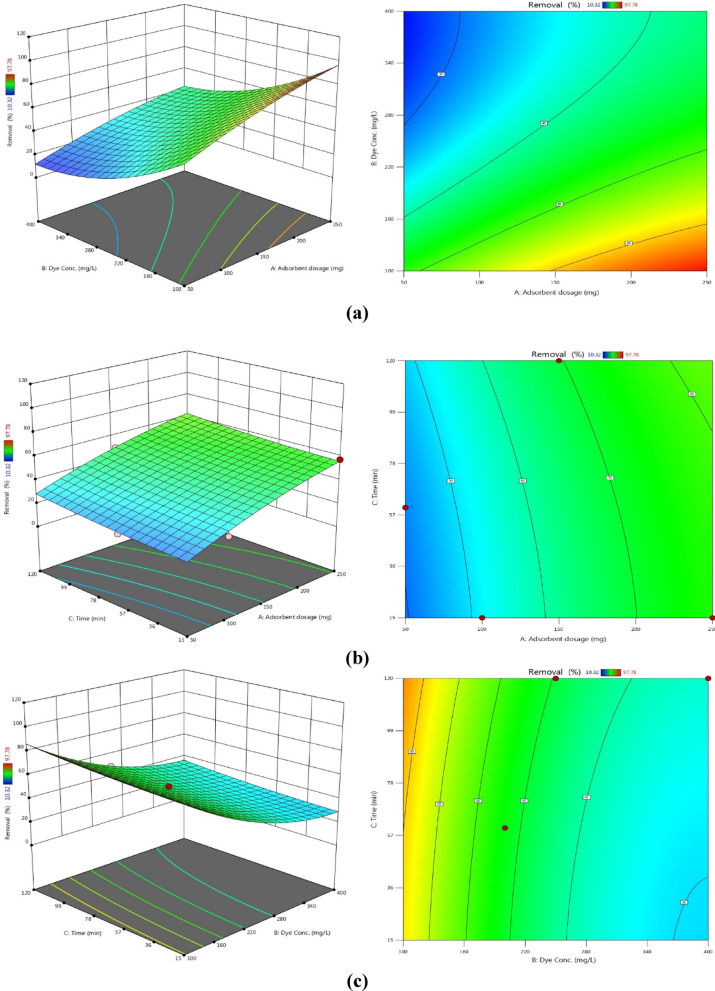
The combined impact of process variables (**a**) adsorbent dosage and starting dye concentration, (**b**) AC7-800 dosage and contact time, and (**c**) starting dye concentration and contact time on AB14 dye removal % with the interaction impact of dual factors.

**Fig. 14:**
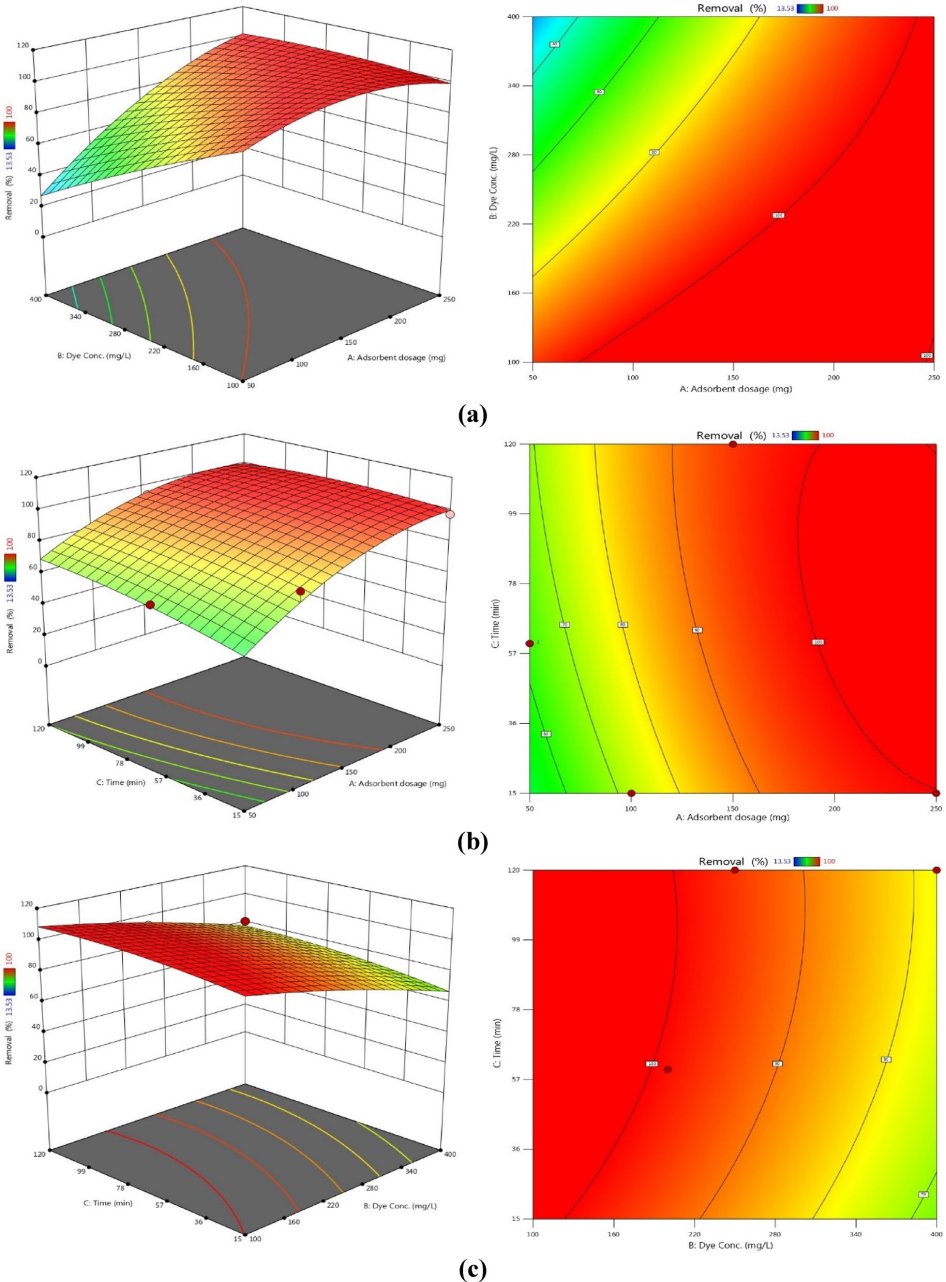
. The combined impact of process variables (**a**) AC7-800 dosage and starting dye concentration, (**b**) AC7-800 dosage and contact time, and (**c**) starting dye concentration and contact time on AY36 adsorption with the interaction impact of dual factors.

Figures 13 and 14 present three-dimensional surface plots illustrating the effects of interactions among three key variables: (A) the dose of AC7-800, (B) the initial dye concentration, and (C) the contact time on the removal efficiencies of AB14 and AY36 dyes.

Figures 13a and a and 14a highlight the interaction between the initial dye concentration and the AC7-800 dosage, demonstrating the significant influence of both factors on dye adsorption. Removal percentages increased with higher adsorbent dosages, attributed to the greater availability of active sites and an expanded adsorbent surface area for adsorption^82,135^. However, as shown in Figs. 13c and 14c, the removal percentage decreased with increasing initial dye concentrations, likely due to the limited active sites on the adsorbent surface at higher adsorbate levels^136^. The results also revealed the rapid initial adsorption rate, driven by the ample surface area and the abundance of unoccupied active sites on the adsorbent^137^. The subsequent decline in removal efficiency was attributed to difficulty accessing the remaining unoccupied sites.

**Fig. 15:**
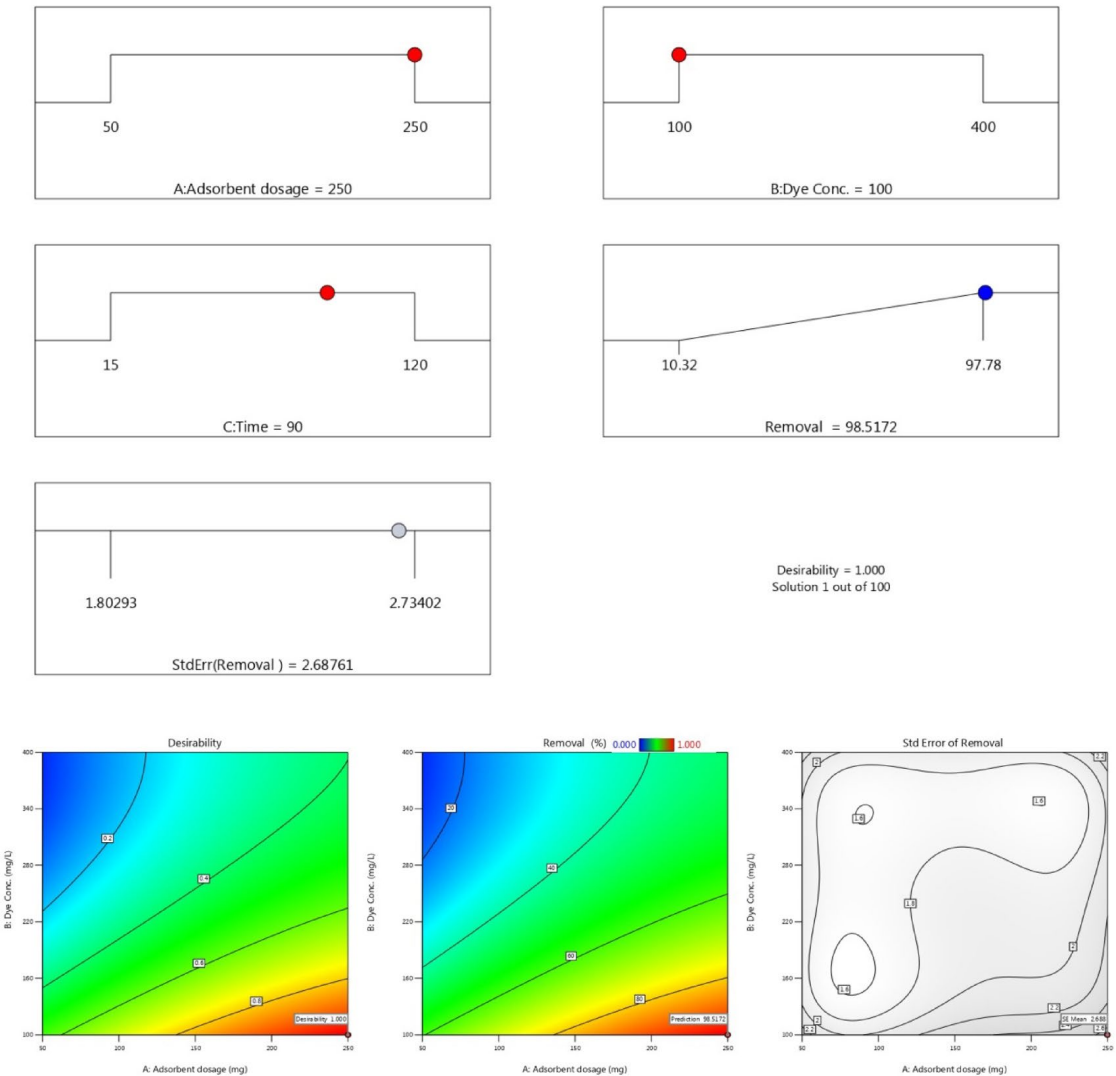
Optimum conditions for AB14 dye adsorption as predicted by the RSM method.

**Fig. 16:**
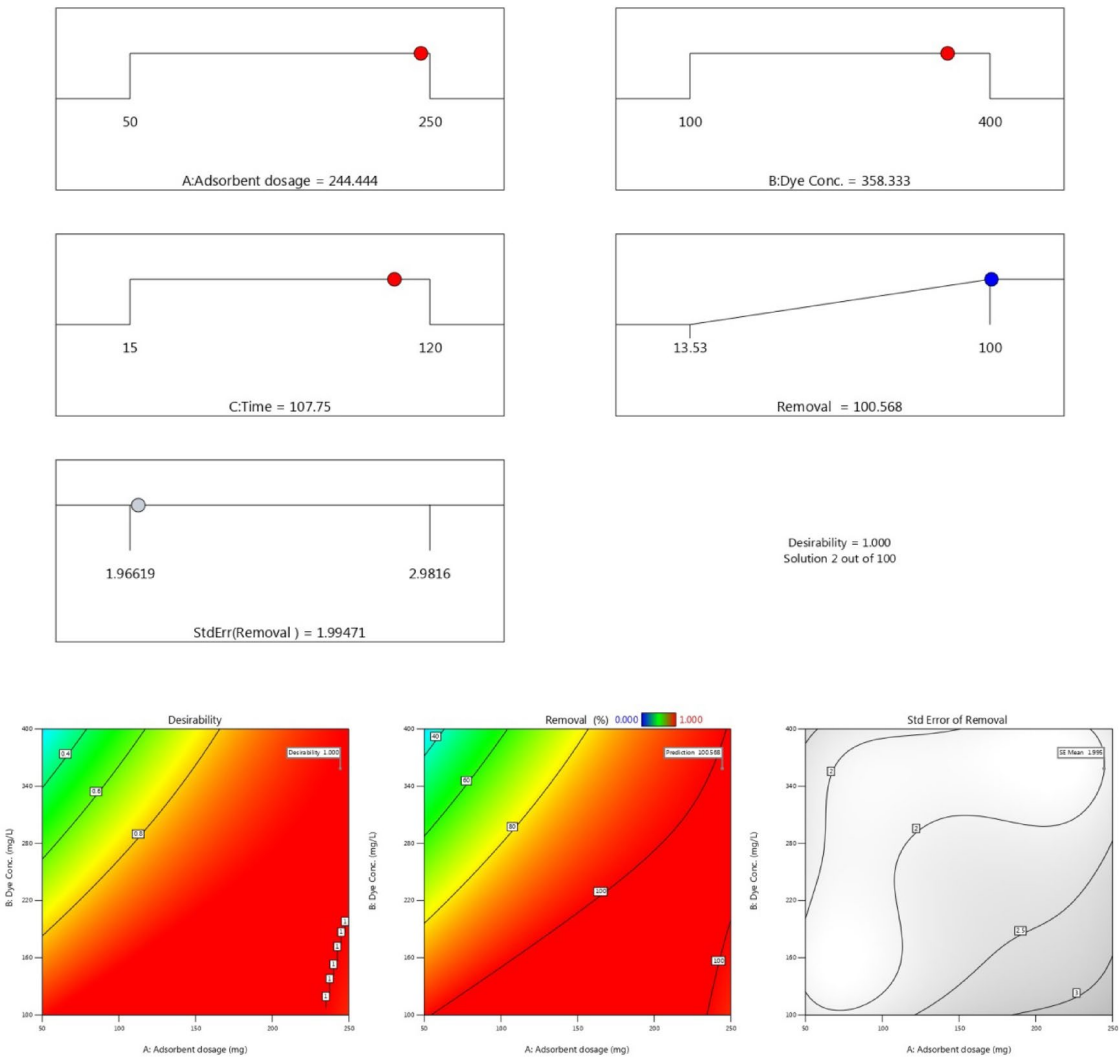
Optimum conditions for AY36 dye adsorption as predicted by the RSM method.

Additional statistical design calculations were performed under identical experimental conditions to further validate and optimize the mathematical model. Figures 15 and 16 show that the maximum desirability value 1 was achieved. For AB14 dye, the optimal removal efficiency of 98.52% was attained at a contact time of 90 min, an AC7-800 dosage of 250 mg, and an initial dye concentration of 100 mg/L. For AY36 dye, complete removal (100%) was achieved at a contact time of 107.75 min, an AC7-800 dosage of 244.4 mg, and an initial dye concentration of 358.3 mg/L.

### ANN modelling

**Fig. 17:**
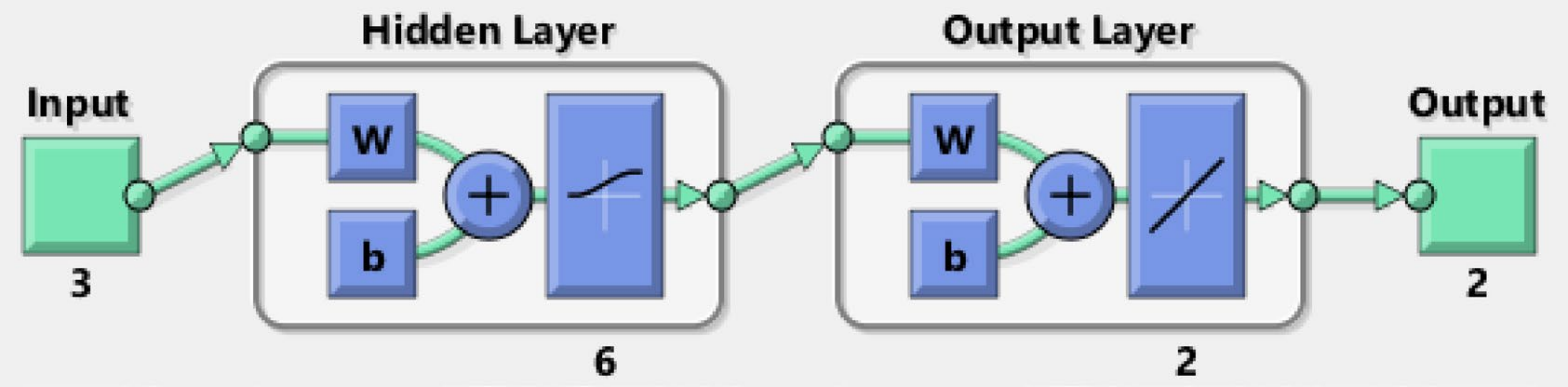
ANN architecture for the removal of AY36 and AB14 dyes by AC7-800.

**Fig. 18:**
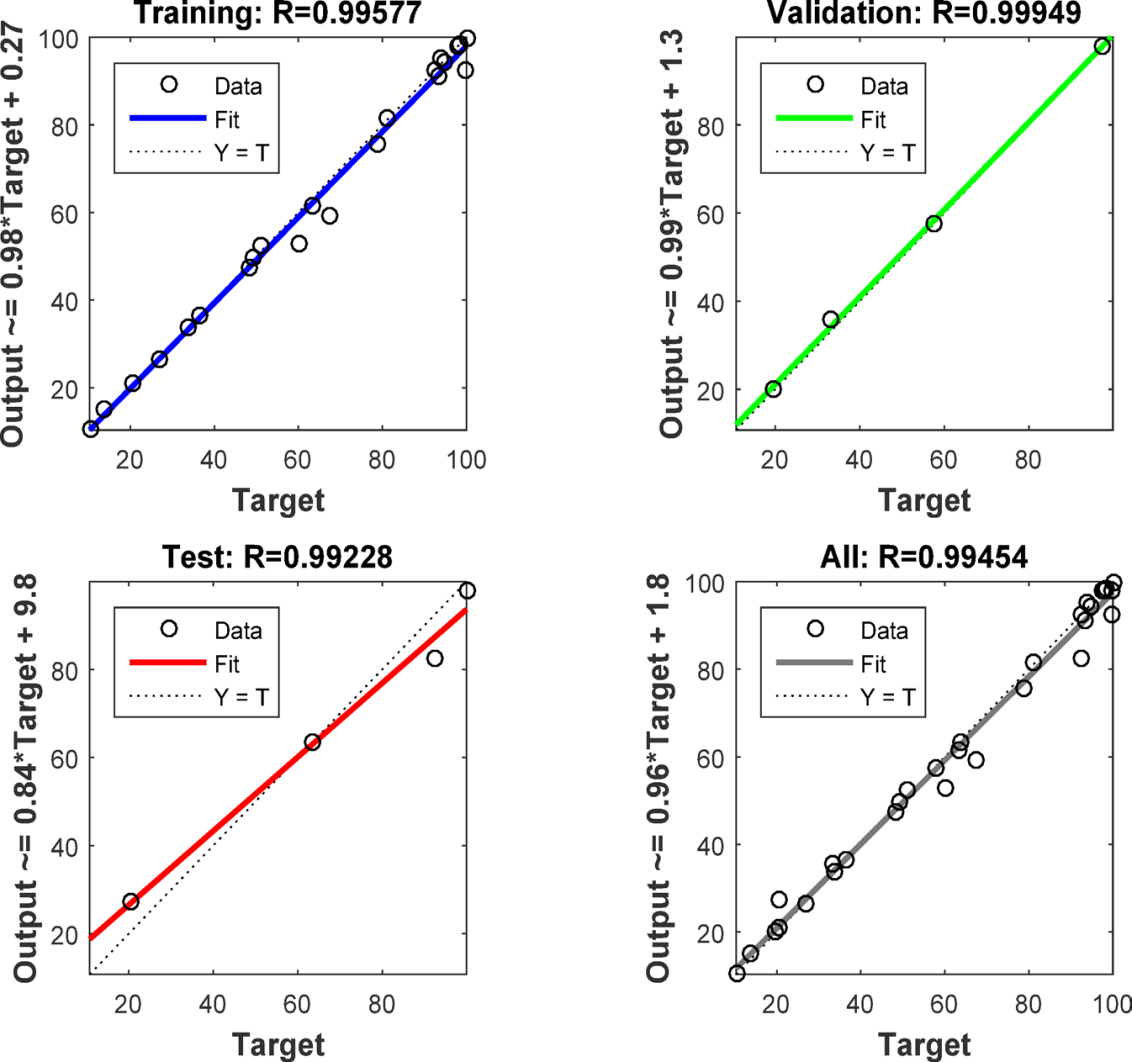
Training, validation, testing, and overall datasets for the LM algorithm.

**Fig. 19:**
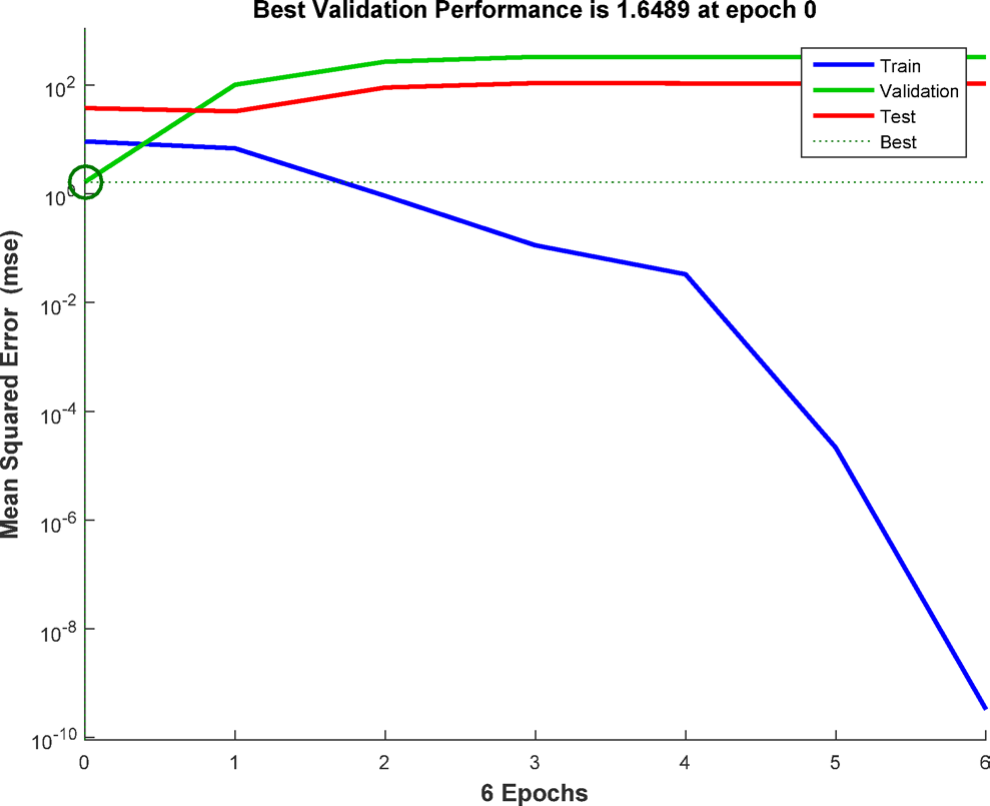
LM algorithm performance.

70:15:15 is the training, validation, and testing ratio for the sample data in this ANN study. The training procedure (backpropagation algorithm) used the ANN performance, the number of validation checks, the performance gradient, and the training epoch number for efficient training. 3-6-2 (3- ILs, 6- HLs, and 2 OL) was the best fit ANN model for the biosorption of AO7 and AB14 dyes to AC7-800 as represented in Fig. 17. The high *R*^2^ and the low MSE error values were displayed by regression plots in Fig. 18. The *R*^2^ for training, validation, testing, and overall were 0.9958, 0.9995, 0.9923, and 0.9945, respectively. The MSE value was 3.28e-10. Although there were some outliers, the ANN model was perfect for testing and validation. The adsorbent dosage of AC7-800 (mg), time (min), and initial concentrations of AY36 and AB14 dyes (mg/L) were the 3 input variables. The removal % of AY36 and AB14 dyes was the two output variables. The input and output variables were specified in the same system. The best ANN possessed 6 neurons in the hidden layer. Log-Sigmoid (log-sig) for the hidden layer and purely for the output layer were the activation functions of the optimal ANN. The MSE error vs. the epoch number for the optimized ANN model is shown in Fig. 19. The training process stopped after 0 epochs for the best ANN, showing that the ANN training model was flawlessly performed at 0 for modelling the adsorption process^138^.

### AC7-800 renewal achievement

**Fig. 20:**
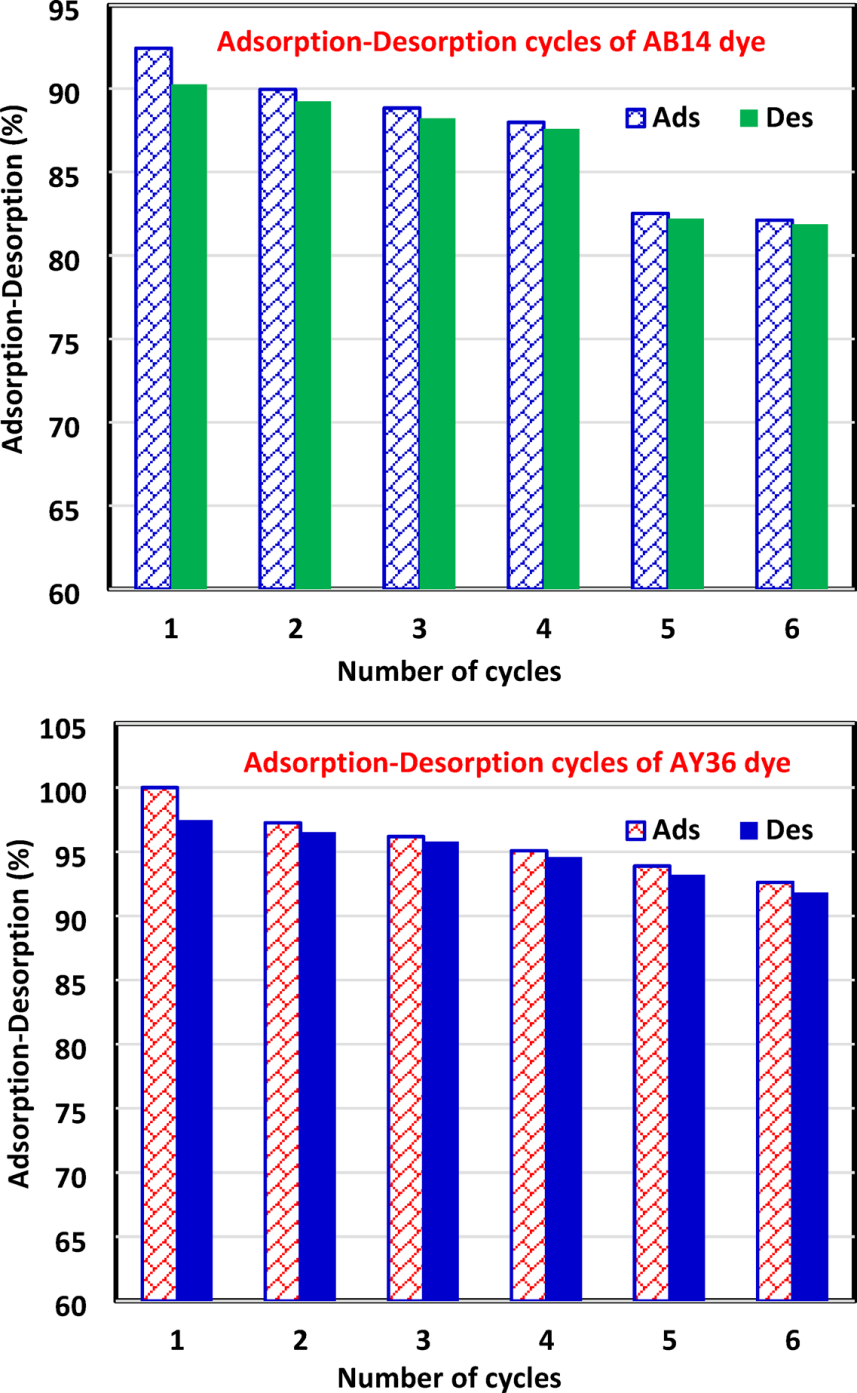
Renewal study of AB14 and AY36 dyes adsorption-desorption by AC7-800 adsorbent using dye *C*_0_ (100 mg/L) and 2.50 g/L AC7-800 dose at room temperature.

Renewable tests were conducted to study the feasibility and reusability of the AC7-800 adsorbent for AB14 and AY36 dye removal. A 0.1 M NaOH solution was employed as the elution medium to desorb the dyes from AC7-800, and dye concentrations were subsequently measured. The adsorbent was then reactivated using 0.1 M HCl. Results indicated that dye desorption decreased with additional regeneration cycles (Fig. 20). Six adsorption/desorption cycles were performed using regenerated AC7-800, showing that adsorption and desorption levels remained nearly constant across cycles. After six cycles, the adsorption and desorption efficiencies decreased by approximately 10.30–8.38% and 7.38–5.65% for AB14 and AY36 dyes, respectively. These results suggest that AC7-800 is a durable adsorbent for removing B14 and AY36 dyes from water (Fig. 20).

### Adsorption mechanism of AB14 and AY36 dyes by AC7-800

**Fig. 21:**
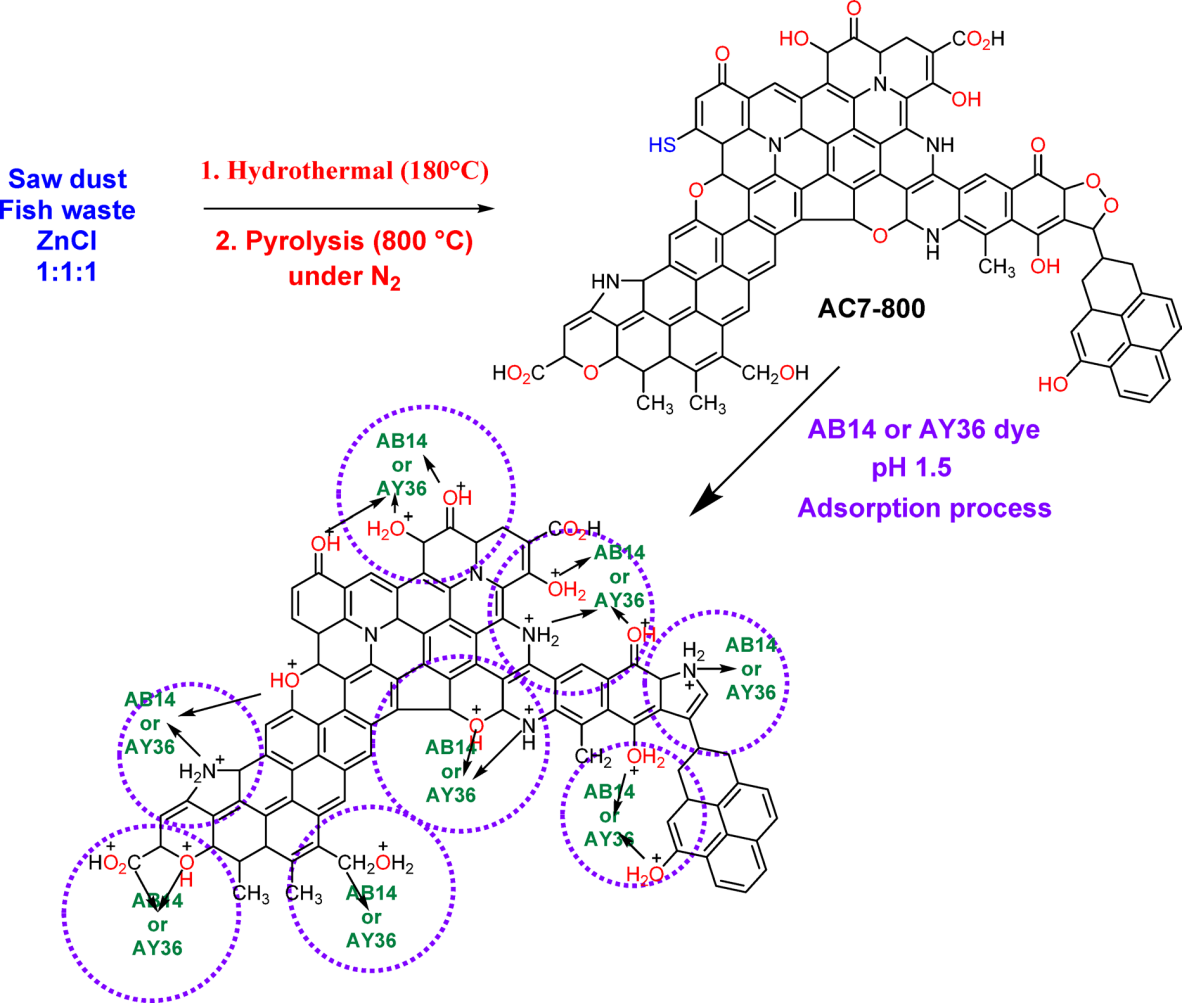
A probable mechanism for the AB14 and AY36 dyes adsorption onto the AC7-800.

The probable mechanism for the removal of the AB14 and AY36 dyes by AC7-800 was explained in Fig. 21. After the pyrolysis of the hydrothermal product of (Fish waste/ZnCl_2_/sawdust in water) at 800 °C, many functional groups were formed on the adsorbent (AC7-800) surface like allene C= C = C, ketamine C = C= N, amide N–H, hydroxyl O–H, and C–N groups. The mechanism of the removal of AB14 and AY36 dyes by AC7-800 in an acidic medium may be achieved via physical interaction due to electrostatic interaction between the positive hydrogen ions in the bulk solution and the nitrogen and oxygen functional groups on the AC7-800 surface, then surface charge became positive; subsequently, electrostatic interaction occurred between the positively charged surface and the anionic dyes species.

## Conclusion

With the use of hydrothermal and pyrolysis processes, our study has successfully described an economical and ecologically responsible method for creating self-doping activated carbons at 800 °C. The prepared AC7-800 showed significant adsorption capabilities for AB14 (*Q*_*e*_ = 107.53 mg g^–1^) and AY36 (*Q*_*e*_=263.16 mg g^–1^) dyes upon reaching equilibrium. Additionally, a concentration of 0.5 g L^–1^ of AC7-800 efficiently absorbed 54.65% and 87.33% of 100 mg L^–1^ of AB14 and AY36 dyes, respectively, within 10 min. Furthermore, nitrogen atoms were effectively introduced into the activated carbon framework, resulting in a nitrogen content of 13.73% following the carbonization process. The AC7-800 material exhibited a specific surface area of 437.51 m^2^ g^–1^, achieved through micropores with a mean pore diameter of 2.0133 nm. Research on the surface chemistry of the AC7-800 revealed that the acidic properties were more prevalent than the essential properties. AC7-800 has a substantial specific surface area, elevated nitrogen content, and contains micro-porous elements. The optimal pH value for removing AB4 and AY36 dyes was 1.5, resulting in elimination efficiencies of 63.29 and 85.86%, respectively. The *Q*_*m*_ values obtained from the LIM process were 107.53 mg g^–1^ for the AB14 dye and 263.16 mg g^–1^ for the AY36 dye. The adsorption kinetics of both AB14 and AY36 dyes onto AC7-800 were most accurately characterized using a PSOM model. This suggests that the adsorption process of both dyes onto AC7-800 is mainly dominated by chemisorption. The adsorption data were well described by LIM. This technique effectively addresses the problems associated with the disposal of dye effluent by converting waste materials such as fish waste (which contains 60% protein) and sawdust into usable resources. The removal % of AB14 and AY36 dyes by AC7-800 was predicted using the D-optimal RSM and ANN models. The ANN model successfully predicted the removal % of AB14 and AY36 dyes compared the RSM model, and it was highly applicable via one hidden layer containing 6 neurons.

## Supplementary Information

Below is the link to the electronic supplementary material.

Supplementary Material 1.

## Data Availability

The corresponding author of the research can provide the datasets used in this study for review upon request.
