# Supplementary material for: Modelling of acid brown 14 and acid yellow 36 dyes adsorption from water by self-nitrogen-doped activated carbon
Source: Sci Rep. 2025 Aug 18;15:30211. doi: 10.1038/s41598-025-14124-7 (PMC12361383; doi:10.1038/s41598-025-14124-7)
Supplement: Supplementary file 1 — Supplementary Material 1. [file 41598_2025_14124_MOESM1_ESM.docx]

**Supplementary materials**

**Modelling of Acid Brown 14 and Acid Yellow 36 dyes adsorption from water by self-nitrogen doped activated carbon**

Mohamed A. El-Nemr^1,2^, Mohamed A. Hassaan^3^, Murat Yılmaz^4^, Ahmed El Nemr^3^*

^1^Department of Chemical Engineering, Faculty of Engineering, Minia University, Minia 61519, Egypt

^2^The Higher Canal Institute of Engineering and Technology, Al Salam 1 - Abu Bakr Al Siddiq Street, Suez, Egypt

^3^Environment Division, National Institute of Oceanography and Fisheries (NIOF), Kayet Bey, Elanfoushy, Alexandria, Egypt

^4^Osmaniye Korkut Ata University, Bahçe Vocational School, Department of Chemistry and Chemical Processing Technologies, Osmaniye, 80000, Türkiye

E-mail: [mohamedelnemr1992@yahoo.com](mailto:mohamedelnemr1992@yahoo.com) (M.A. El-Nemr), [muratyilmaz@osmaniye.edu.tr](mailto:muratyilmaz@osmaniye.edu.tr) (M. Yılmaz), [mhss95@mail.com](mailto:mhss95@mail.com) (M.A. Hassaan)

*Corresponding author: [ahmedmoustafaelnemr@yahoo.com](mailto:ahmedmoustafaelnemr@yahoo.com); [ahmed.m.elnemr@gmail.com](mailto:ahmed.m.elnemr@gmail.com)

| 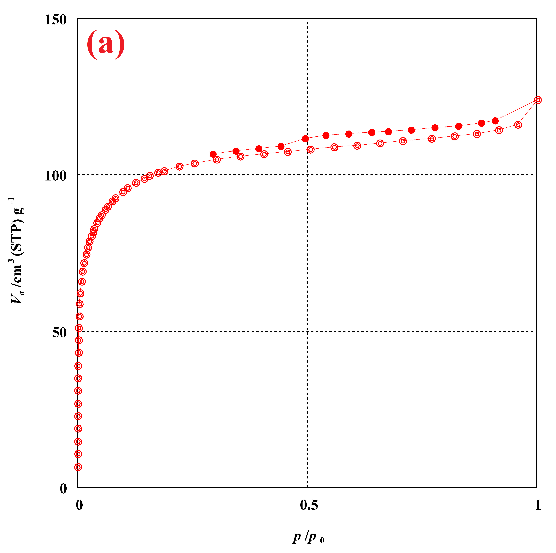 | 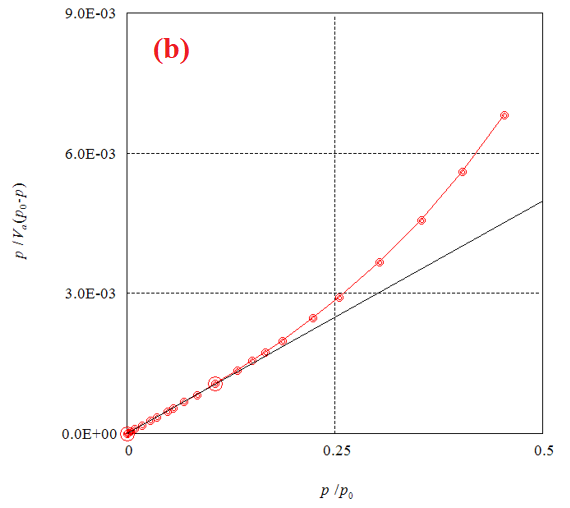 |
| --- | --- |
| 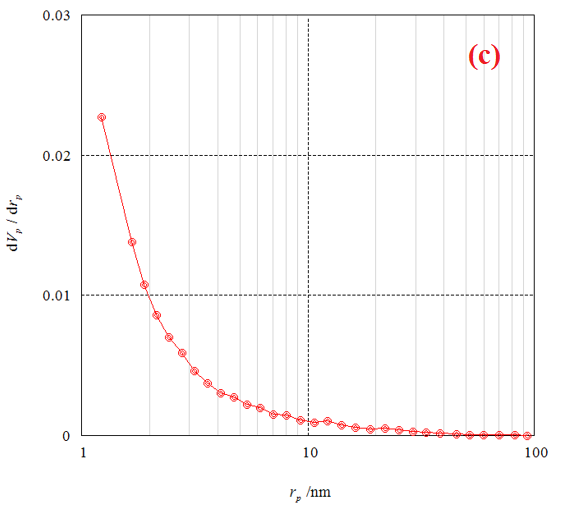 | 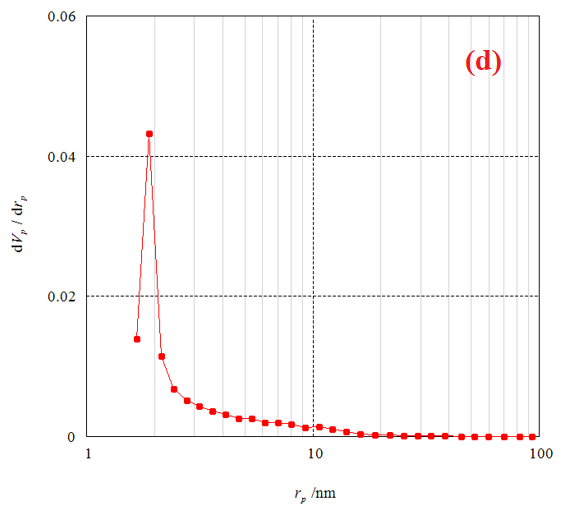 |
| 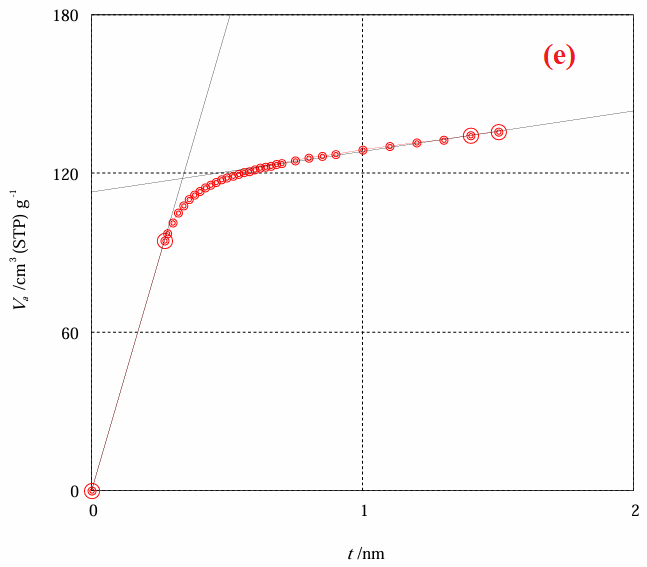 | 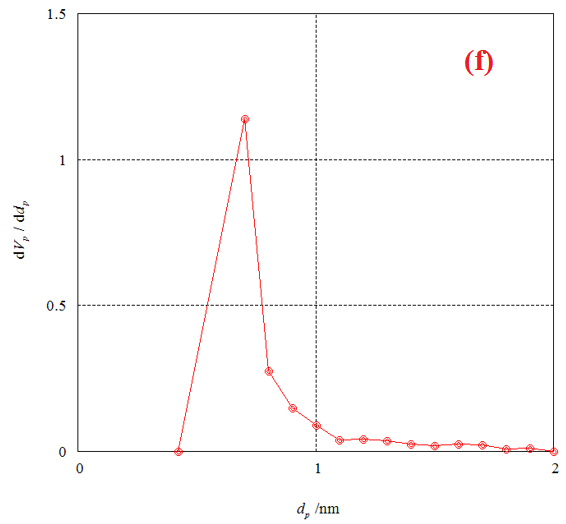 |

**Figure S1**. (a) Adsorption–desorption of AC7-800; (b) BET plot of AC7-800; (c) BJH plot by adsorption of AC7-800; (d) BJH plot by desorption of AC7-800; (e) *t*-plot of AC7-800; (f) MP plot of AC7-800.

|  |  |
| --- | --- |
|  |  |

**Figure S2**. The profiles of (a) LIM (b) FIM (c) TIM, and (d) HIM for AB14 dye of initial concentration (100 - 400 mg L^–1^) on AC7-800 dosages (0.50 - 2.50 g L^−1^) at 25±2 °C, contact time: 120 min).

|  |  |
| --- | --- |
|  |  |

**Figure S3**. The profiles of (a) LIM (b) FIM (c) TIM, and (d) HIM for AY36 dye of initial concentration (100 - 400 mg L^–1^) on AC7-800 dosages (0.50 - 2.50 g L^−1^) at 25±2 °C, contact time: 120 min).

|  |  |
| --- | --- |
|  |  |
|  | |

**Figure S4**. The plot of (a) PFOM, (b) PSOM, (c) EM, (d) IPDM, and (e) FDM of adsorption of AB14 dye by AC7-800 adsorbent (*C*_0_ = 100 - 400 mg L^-1^, AC7-800 = 5.0 g L^−1^, Temp. = 25±2 °C).

|  |  |
| --- | --- |
|  |  |
|  | |

**Figure S5**. The plot of (a) PFOM (b) PSOM (c) EM (d) IPDM (e) FDM of adsorption of AY36 dye by AC7-800 adsorbent (C_0_ = 100 - 400 mg L^-1^, AC7-800 = 5.0 g L^−1^, Temp. = 25±2 °C).

| 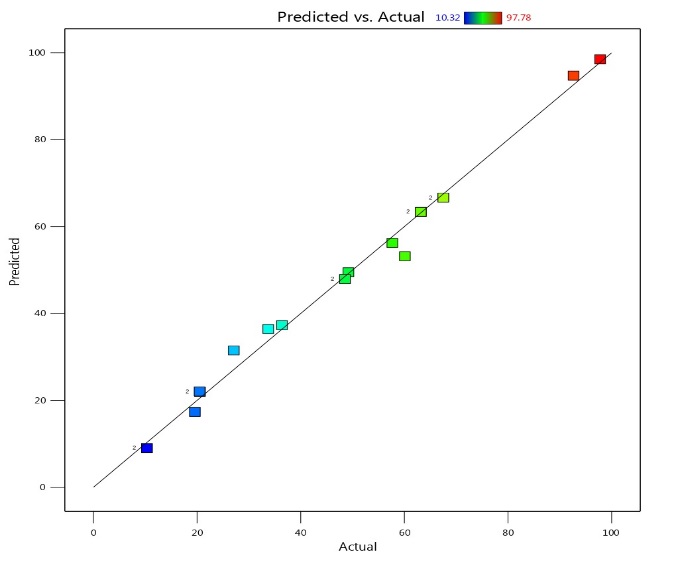 | 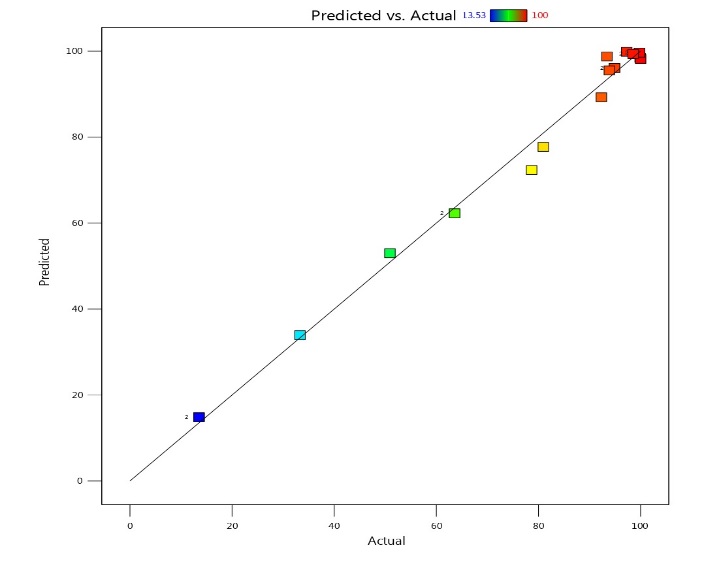 |
| --- | --- |
| **(a)** | **(b)** |

**Figure S6**. Plots between the experimental and predicted data for (a) AB14 dye adsorption, and (b) AY36 dye adsorption.

**Table S1**. Experimental design and RSM and ANN removal prediction percentage for adsorption of AB14 and AY36 dyes on AC7-800.

|  | **Factor 1** | **Factor 2** | **Factor 3** | **Response** | | | **Response** | | |
| --- | --- | --- | --- | --- | --- | --- | --- | --- | --- |
|  |  |  |  | **AB14 dye removal %** | | | **AY36 dye removal %** | | |
|  | **A: AC7-800 dosage** | **B: Dye Conc.** | **C: Time** | **Experiment** | **RSM** | **ANN** | **Experiment** | **RSM** | **ANN** |
| Run |  |  |  |  | **Predicted** | **Predicted** |  | **Predicted** | **Predicted** |
|  | mg | mg/L | min | % | % | *%* | *%* | *%* | *%* |
| 1 | 50 | 250 | 60 | 20.54 | 21.99 | 20.714 | 63.59 | 62.28 | 63.35 |
| 2 | 50 | 400 | 15 | 10.32 | 9.03 | 10.237 | 13.53 | 14.83 | 13.75 |
| 3 | 200 | 400 | 45 | 36.39 | 37.32 | 36.405 | 92.33 | 89.28 | 92.10 |
| 4 | 100 | 100 | 15 | 67.52 | 66.68 | 67.787 | 94.89 | 96.07 | 94.69 |
| 5 | 150 | 400 | 120 | 33.73 | 36.43 | 33.224 | 80.96 | 77.71 | 80.94 |
| 6 | 250 | 250 | 15 | 57.68 | 56.25 | 57.759 | 97.28 | 99.85 | 97.28 |
| 7 | 100 | 250 | 15 | 27.09 | 31.49 | 27.011 | 78.67 | 72.35 | 78.59 |
| 8 | 250 | 400 | 120 | 48.54 | 47.94 | 48.485 | 98.55 | 99.35 | 98.44 |
| 9 | 150 | 250 | 120 | 49.21 | 49.49 | 48.885 | 93.89 | 95.51 | 93.97 |
| 10 | 50 | 400 | 15 | 10.32 | 9.03 | 10.237 | 13.53 | 14.83 | 13.65 |
| 11 | 250 | 400 | 120 | 48.54 | 47.94 | 48.385 | 98.55 | 99.35 | 98.44 |
| 12 | 150 | 200 | 60 | 60.11 | 53.18 | 60.502 | 93.42 | 98.76 | 93.52 |
| 13 | 250 | 100 | 30 | 92.66 | 94.74 | 92.527 | 99.93 | 98.48 | 99.86 |
| 14 | 100 | 400 | 60 | 20.49 | 22.08 | 20.421 | 50.92 | 52.98 | 50.83 |
| 15 | 50 | 400 | 120 | 19.58 | 17.36 | 19.565 | 33.31 | 33.93 | 33.21 |
| 16 | 100 | 100 | 15 | 67.52 | 66.68 | 67.787 | 94.89 | 96.07 | 94.78 |
| 17 | 50 | 250 | 60 | 20.54 | 21.99 | 20.714 | 63.59 | 62.28 | 63.45 |
| 18 | 50 | 100 | 120 | 63.19 | 63.39 | 63.118 | 99.79 | 99.65 | 99.54 |
| 19 | 250 | 100 | 90 | 97.78 | 98.52 | 93.367 | 100 | 98.18 | 99.95 |
| 20 | 50 | 100 | 120 | 63.19 | 63.39 | 57.0177 | 99.79 | 99.65 | 99.54 |

**Table S2**. D-optimal design for AB14 dye adsorption using ANOVA and model fit summary.

| **Source** | **Value** | **Sum of Squares** | **df** | **Mean Square** | **F-value** | **p-value** | **Remarks** | **source** | **SD** | **R²** | **Adjusted R²** | **Predicted R²** | **PRESS** | **Remarks** |
| --- | --- | --- | --- | --- | --- | --- | --- | --- | --- | --- | --- | --- | --- | --- |
| **Model** | - | 12425.02 | 9 | 1380.56 | 138.36 | < 0.0001 | significant | - | - | - | - | - | - | - |
| A- AC7-800 dosage | - | 3294.49 | 1 | 3294.49 | 330.17 | < 0.0001 | - | - | - | - | - | - | - | - |
| B-Dye Conc. | - | 6441.48 | 1 | 6441.48 | 645.57 | < 0.0001 | - | - | - | - | - | - | - | - |
| C-Time | - | 148.32 | 1 | 148.32 | 14.86 | 0.0032 | - | - | - | - | - | - | - | - |
| AB | - | 27.55 | 1 | 27.55 | 2.76 | 0.1275 | - | - | - | - | - | - | - | - |
| AC | - | 2.39 | 1 | 2.39 | 0.2397 | 0.6350 | - | - | - | - | - | - | - | - |
| BC | - | 0.9538 | 1 | 0.9538 | 0.0956 | 0.7635 | - | - | - | - | - | - | - | - |
| A² | - | 43.03 | 1 | 43.03 | 4.31 | 0.0646 | - | - | - | - | - | - | - | - |
| B² | - | 488.48 | 1 | 488.48 | 48.96 | < 0.0001 | - | - | - | - | - | - | - | - |
| C² | - | 5.47 | 1 | 5.47 | 0.5485 | 0.4760 | - | - | - | - | - | - | - | - |
| **Residual** | - | 99.78 | 10 | 9.98 |  |  | - | - | - | - | - | - | - | - |
| Lack of Fit | - | 99.78 | 5 | 19.96 |  |  | - | - | - | - | - | - | - | - |
| Pure Error | - | 0.0000 | 5 | 0.0000 |  |  | - | - | - | - | - | - | - | - |
| **Cor Total** | - | 12524.80 | 19 |  |  |  |  | - | - | - | - | - | - | - |
| **SD** | 3.16 | - | - | - | - | - | - | - | - | - | - | - | - | - |
| **Mean** | 45.75 | - | - | - | - | - | - | - | - | - | - | - | - | - |
| **C.V.%** | 6.90 | - | - | - | - | - | - | - | - | - | - | - | - | - |
| **R²** | 0.9920 | - | - | - | - | - | - | - | - | - | - | - | - | - |
| **Adjusted R²** | 0.9849 | - | - | - | - | - | - | - | - | - | - | - | - | - |
| **Predicted R²** | 0.9722 | - | - | - | - | - | - | - | - | - | - | - | - | - |
| **Adeq Precision** | 40.0629 | - | - | - | - | - | - | - | - | - | - | - | - | - |
| **Linear** |  |  |  |  |  |  |  |  | 6.60 | 0.9443 | 0.9339 | 0.9209 | 990.71 |  |
| 2FI |  |  |  |  |  |  |  |  | 7.14 | 0.9471 | 0.9226 | 0.8927 | 1344.26 |  |
| **Quadratic** |  |  |  |  |  |  |  |  | **3.16** | **0.9920** | **0.9849** | **0.9722** | **348.79** | **Suggested** |
| Cubic |  |  |  |  |  |  |  |  | 0.0000 | 1.0000 | 1.0000 |  | * | Aliased |

**Table S3**. ANOVA and model fit summary for D-optimal design for AY36 dye adsorption

| **Source** | **Value** | **Sum of Squares** | **df** | **Mean Square** | **F-value** | **p-value** | **Remarks** | **source** | **SD** | **R²** | **Adjusted R²** | **Predicted R²** | **PRESS** | **Remarks** |
| --- | --- | --- | --- | --- | --- | --- | --- | --- | --- | --- | --- | --- | --- | --- |
| **Model** | - | 15746.04 | 9 | 1749.56 | 147.43 | < 0.0001 | significant | - | - | - | - | - | - | - |
| A-Time | - | 3969.74 | 1 | 3969.74 | 334.52 | < 0.0001 | - | - | - | - | - | - | - | - |
| B-Dose | - | 2858.10 | 1 | 2858.10 | 240.85 | < 0.0001 | - | - | - | - | - | - | - | - |
| C-Conc | - | 172.20 | 1 | 172.20 | 14.51 | 0.0034 | - | - | - | - | - | - | - | - |
| AB | - | 2229.99 | 1 | 2229.99 | 187.92 | < 0.0001 | - | - | - | - | - | - | - | - |
| AC | - | 131.17 | 1 | 131.17 | 11.05 | 0.0077 | - | - | - | - | - | - | - | - |
| BC | - | 7.96 | 1 | 7.96 | 0.6704 | 0.4320 | - | - | - | - | - | - | - | - |
| A² | - | 368.15 | 1 | 368.15 | 31.02 | 0.0002 | - | - | - | - | - | - | - | - |
| B² | - | 20.05 | 1 | 20.05 | 1.69 | 0.2228 | - | - | - | - | - | - | - | - |
| C² | - | 24.94 | 1 | 24.94 | 2.10 | 0.1778 | - | - | - | - | - | - | - | - |
| **Residual** | - | 118.67 | 10 | 11.87 |  |  | - | - | - | - | - | - | - | - |
| Lack of Fit | - | 118.67 | 5 | 23.73 |  |  | - | - | - | - | - | - | - | - |
| Pure Error | - | 0.0000 | 5 | 0.0000 |  |  | - | - | - | - | - | - | - | - |
| **Cor Total** | - | 15864.71 | 19 |  |  |  | - | - | - | - | - | - | - | - |
| **SD** | 3.44 | - | - | - | - | - | - | - | - | - | - | - | - | - |
| **Mean** | 78.07 | - | - | - | - | - | - | - | - | - | - | - | - | - |
| **C.V.%** | 4.41 | - | - | - | - | - | - | - | - | - | - | - | - | - |
| **R²** | 0.9925 | - | - | - | - | - | - | - | - | - | - | - | - | - |
| **Adjusted R²** | 0.9858 | - | - | - | - | - | - | - | - | - | - | - | - | - |
| **Predicted R²** | 0.9700 | - | - | - | - | - | - | - | - | - | - | - | - | - |
| **Adeq Precision** | 34.9024 | - | - | - | - | - | - | - | - | - | - | - | - | - |
| **Linear** |  |  |  |  |  |  |  |  | 14.75 | 0.7805 | 0.7393 | 0.6289 | 5886.73 |  |
| 2FI |  |  |  |  |  |  |  |  | 6.98 | 0.9601 | 0.9417 | 0.8921 | 1711.03 |  |
| **Quadratic** |  |  |  |  |  |  |  |  | 3.44 | 0.9925 | 0.9858 | 0.9700 | 475.62 | **Suggested** |
| Cubic |  |  |  |  |  |  |  |  | 0.0000 | 1.0000 | 1.0000 |  | * | Aliased |
